# Supplementary figures and images for: An ER phospholipid hydrolase drives ER-associated mitochondrial constriction for fission and fusion
Source: eLife. 2022 Nov 30;11:e84279. doi: 10.7554/eLife.84279 (PMC9725753; doi:10.7554/eLife.84279)

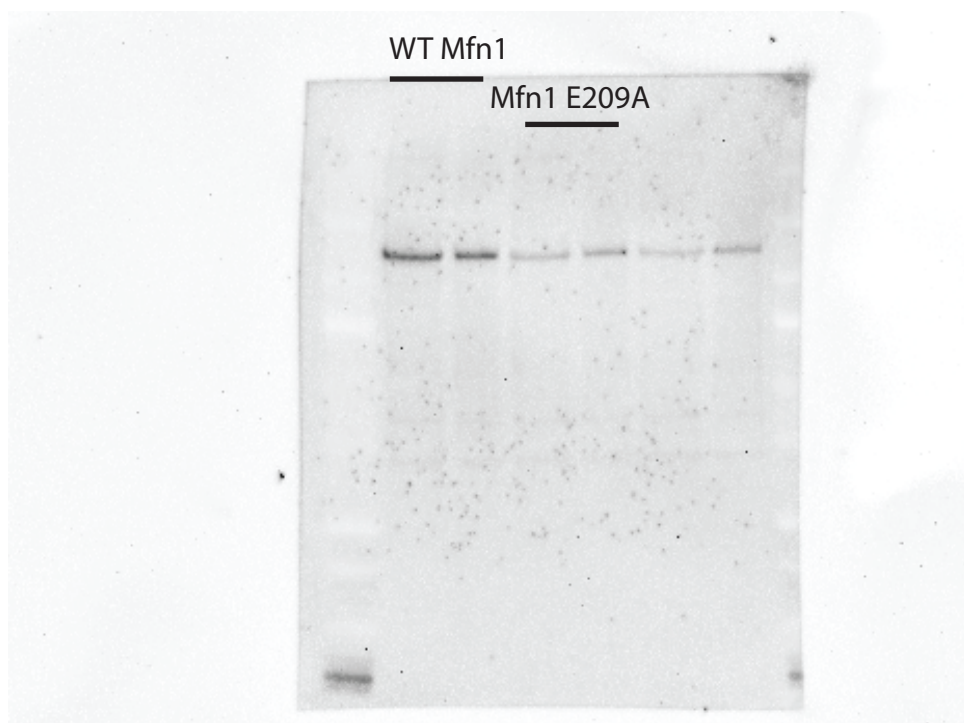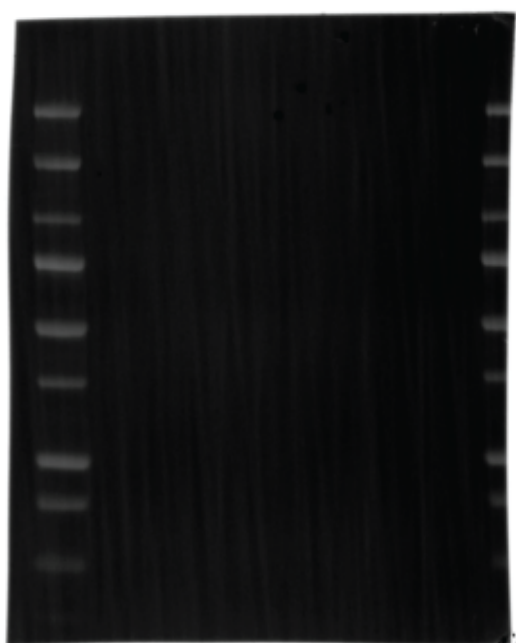

Supplement: Figure 1—source data 1. [file elife-84279-fig1-data1.pdf]

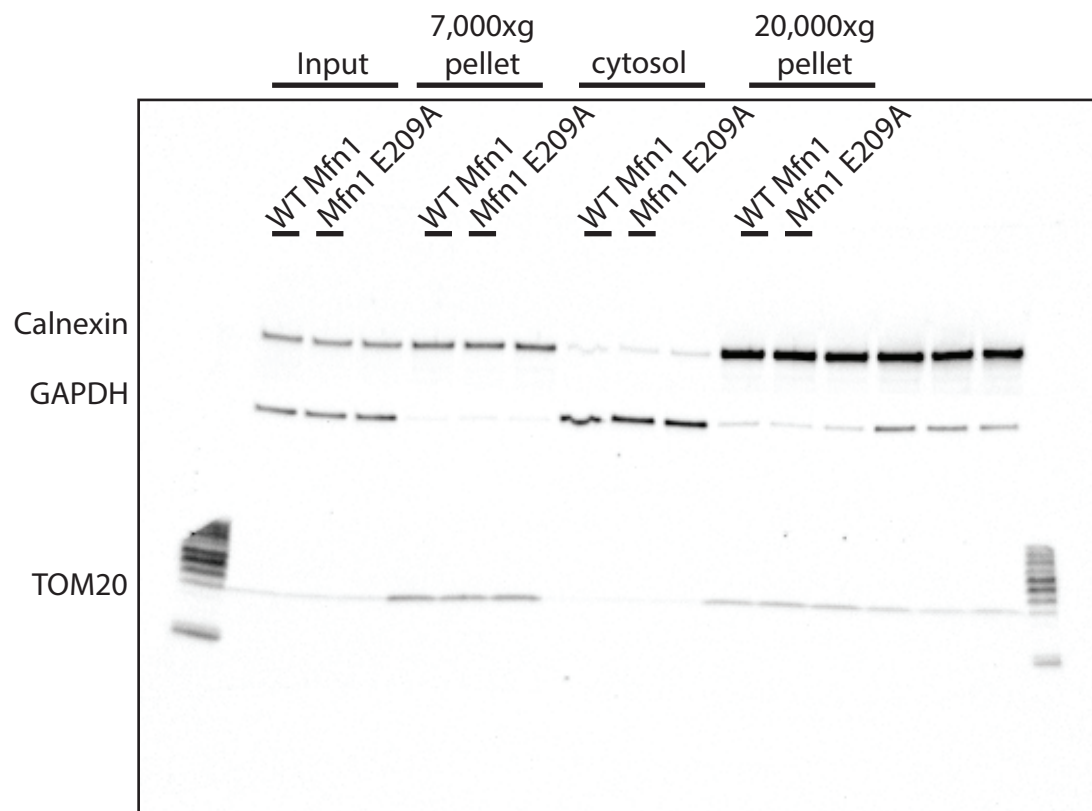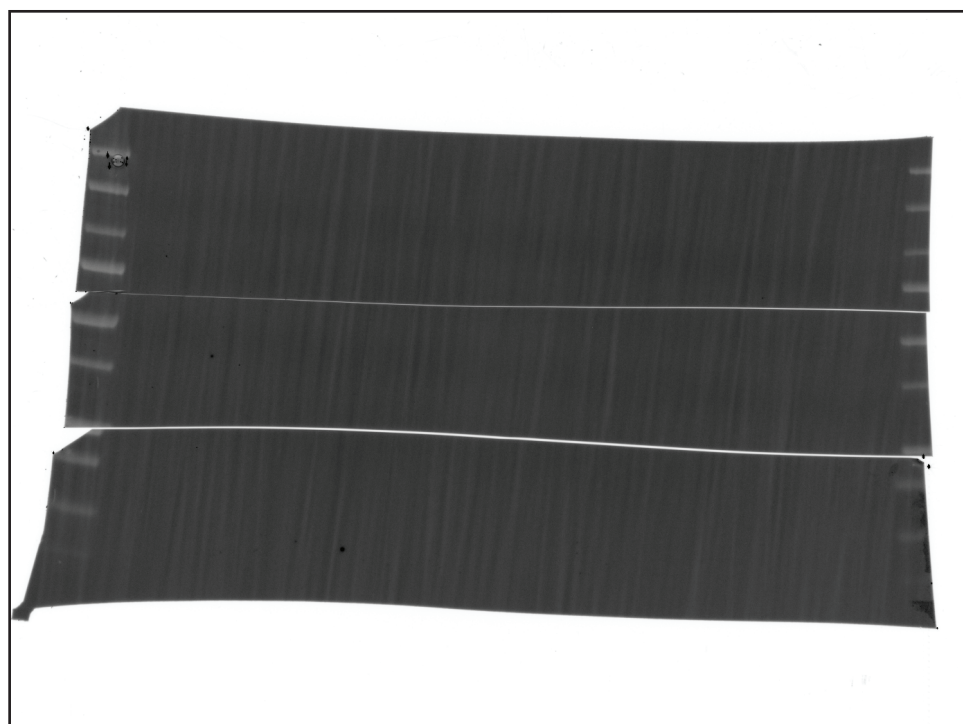

Supplement: Figure 1—source data 2. [file elife-84279-fig1-data2.pdf]

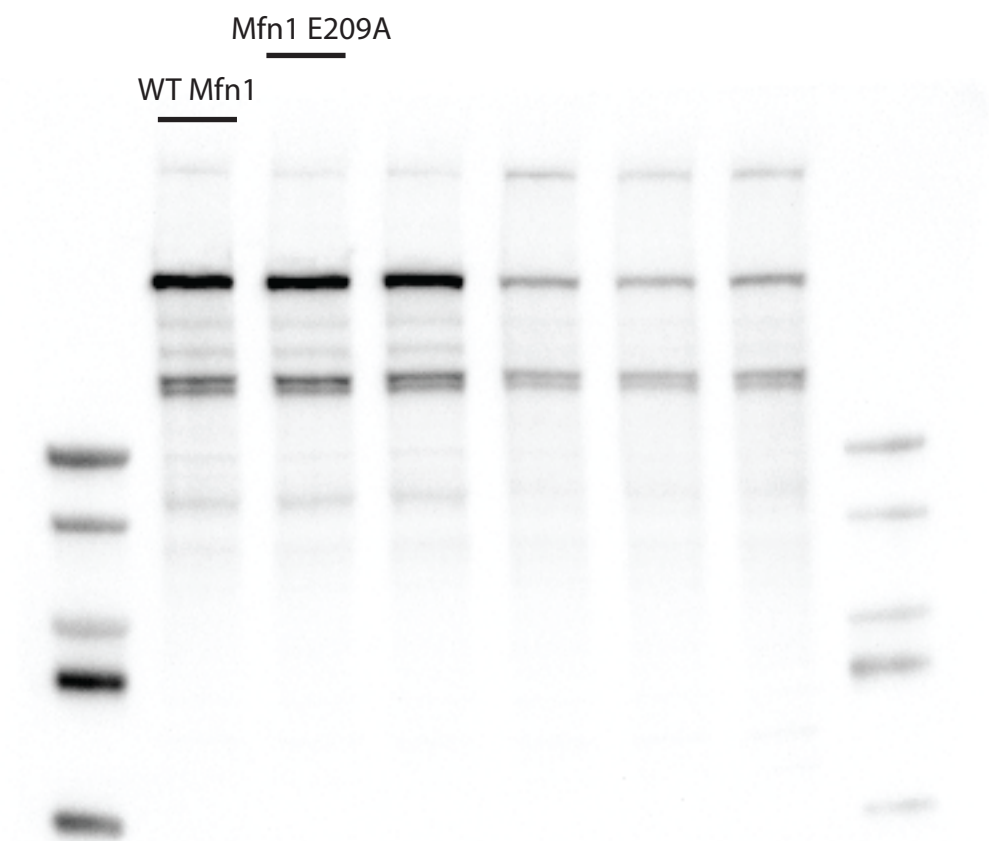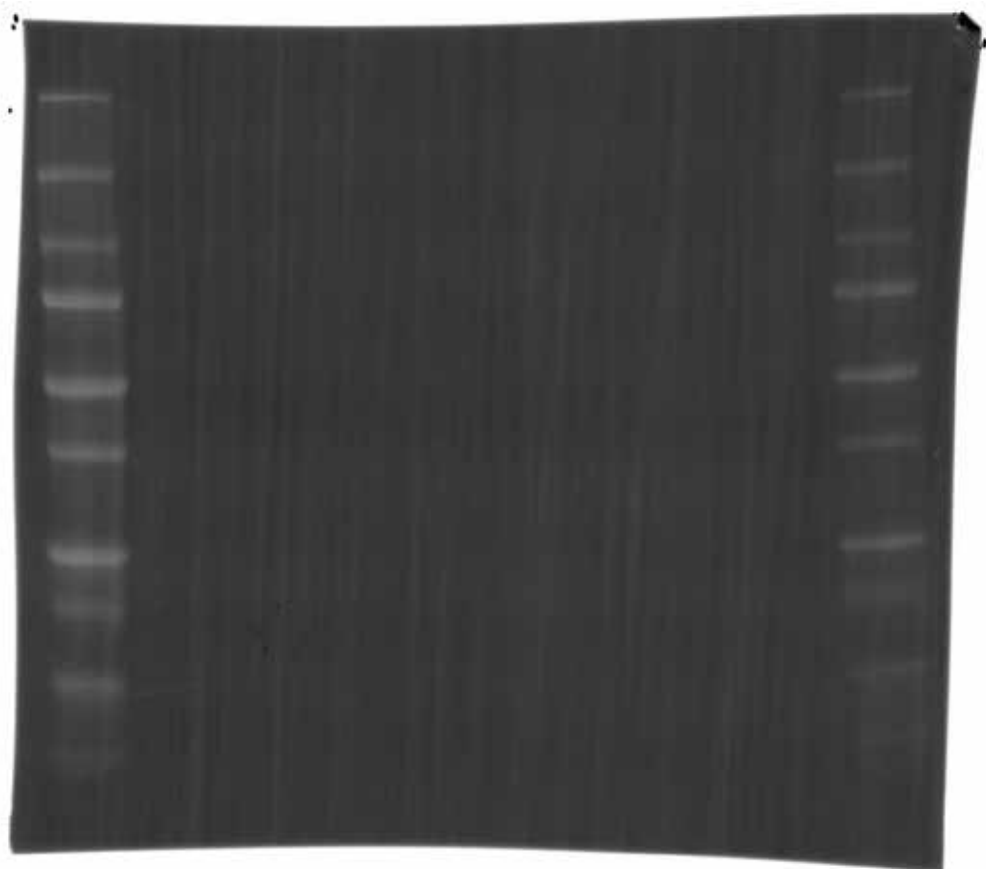

Supplement: Figure 1—source data 3. [file elife-84279-fig1-data3.pdf]

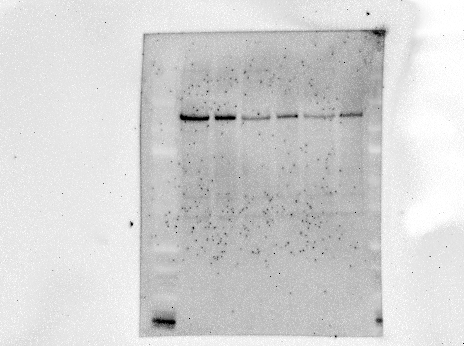

Supplement: Figure 1—source data 5. [file elife-84279-fig1-data5.zip › Figure 1-source data 5.tif]

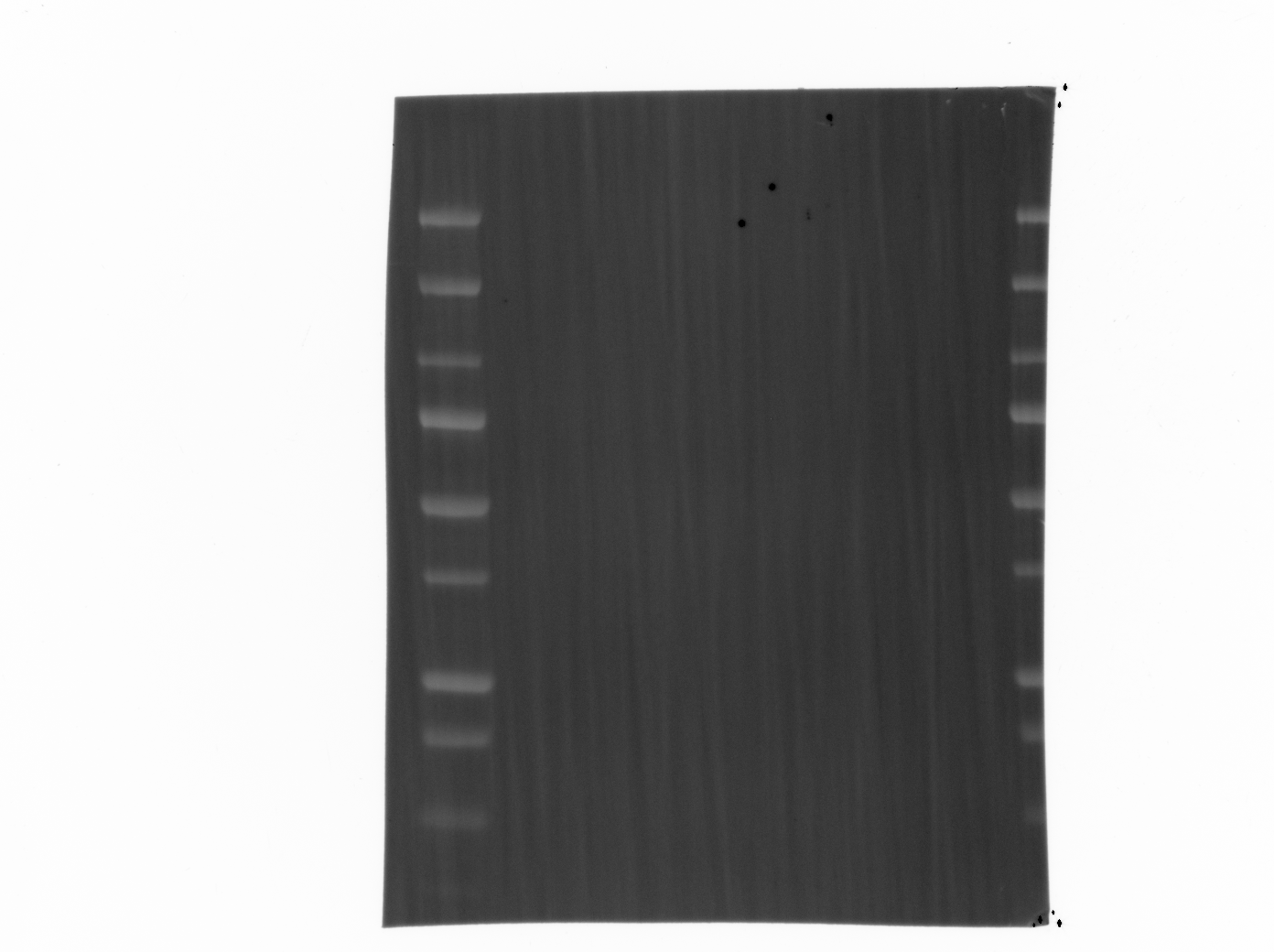

Supplement: Figure 1—source data 6. [file elife-84279-fig1-data6.zip › Figure 1-source data 6.tif]

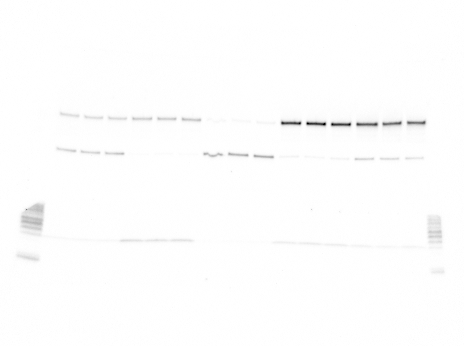

Supplement: Figure 1—source data 7. [file elife-84279-fig1-data7.zip › Figure 1-source data 7.tif]

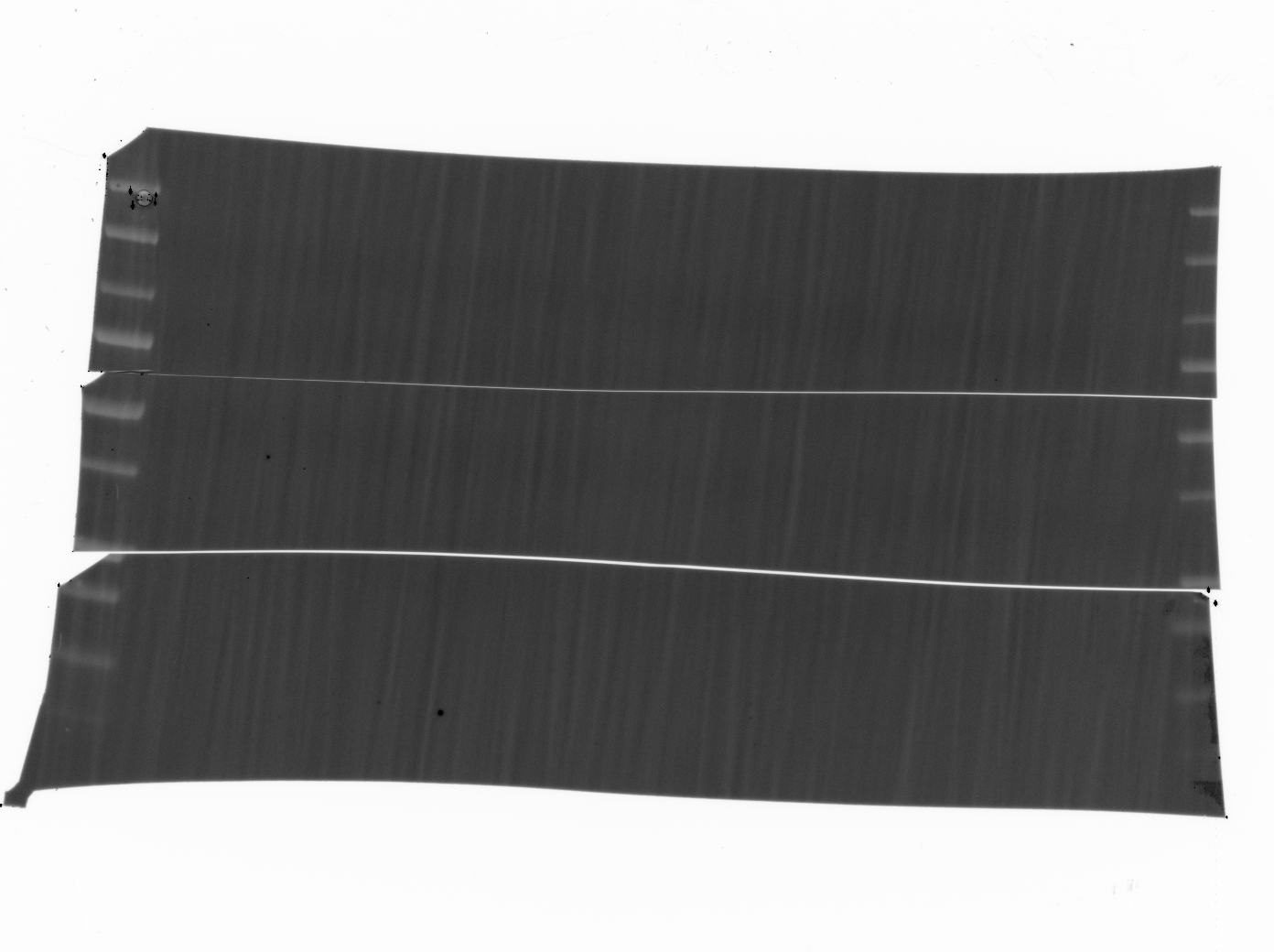

Supplement: Figure 1—source data 8. [file elife-84279-fig1-data8.zip › Figure 1-source data 8.tif]

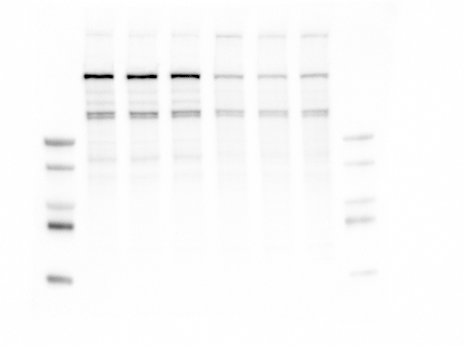

Supplement: Figure 1—source data 9. [file elife-84279-fig1-data9.zip › Figure 1-source data 9.tif]

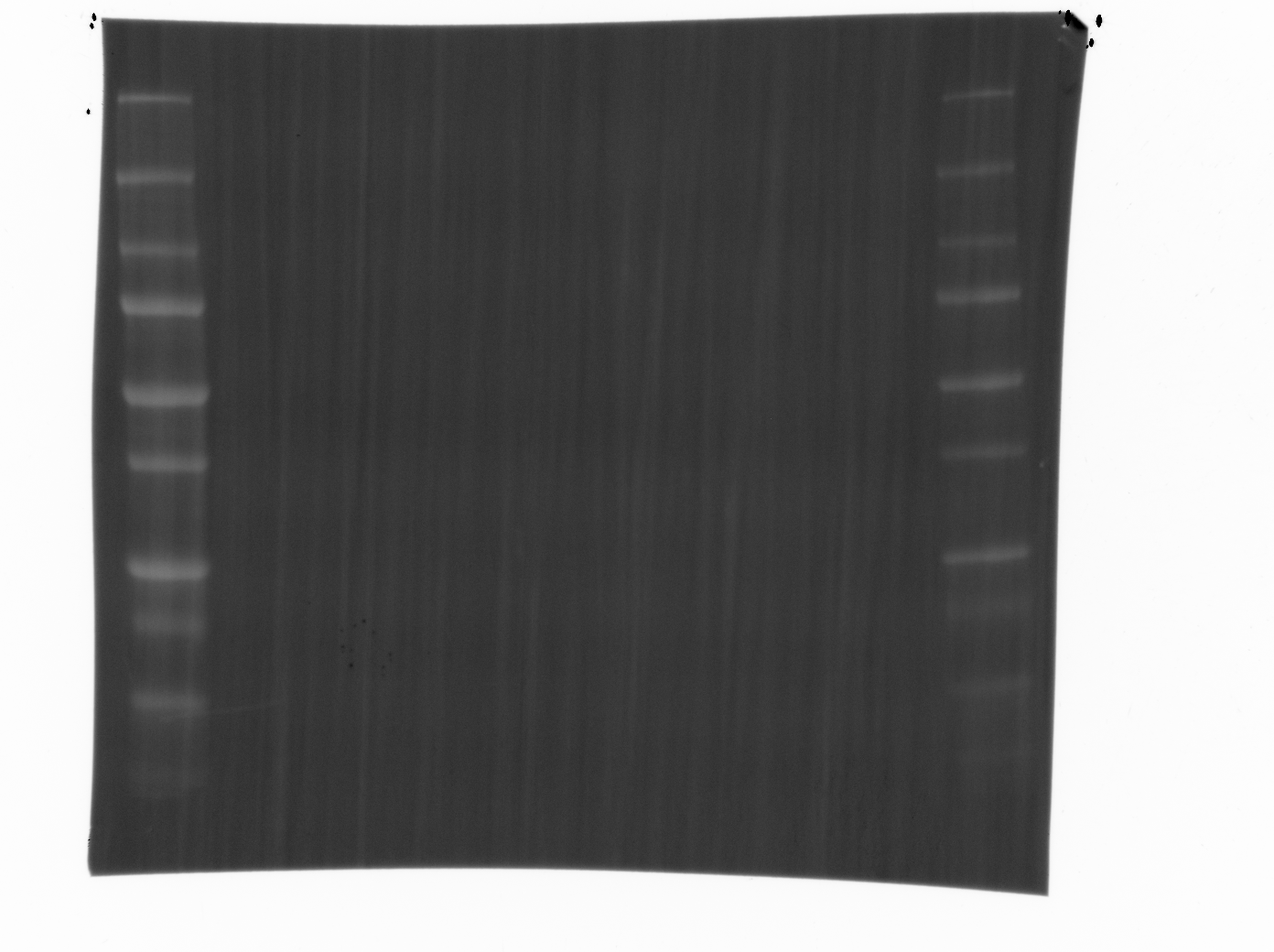

Supplement: Figure 1—source data 10. [file elife-84279-fig1-data10.zip › Figure 1-source data 10.tif]

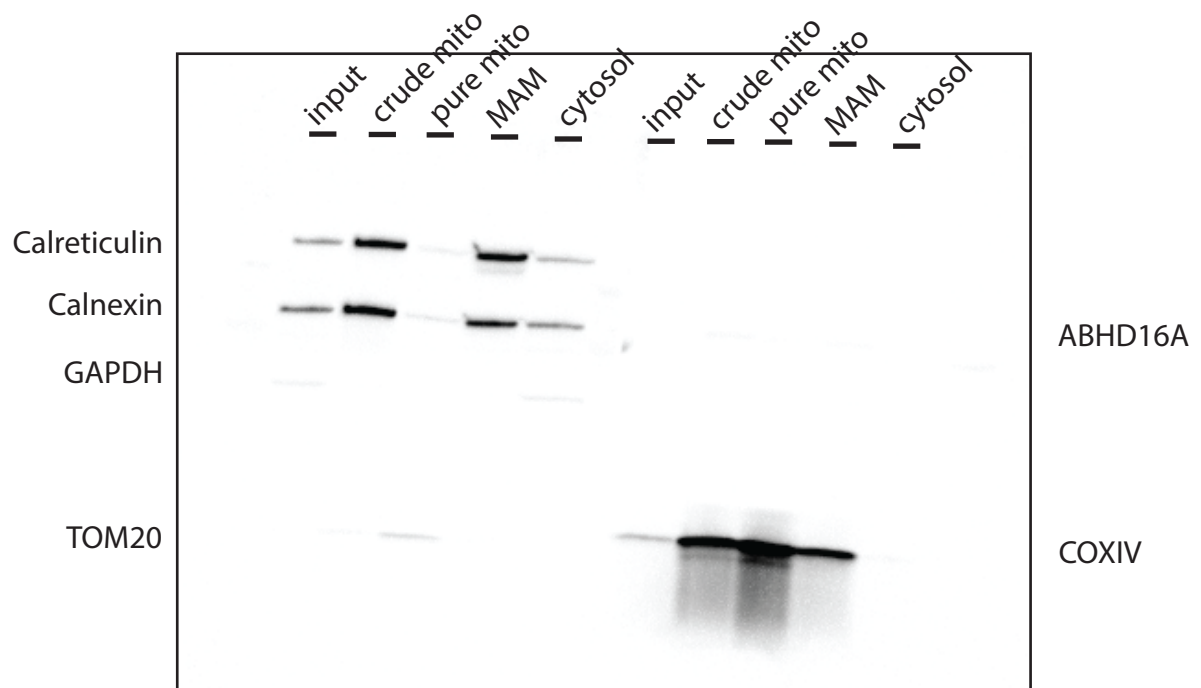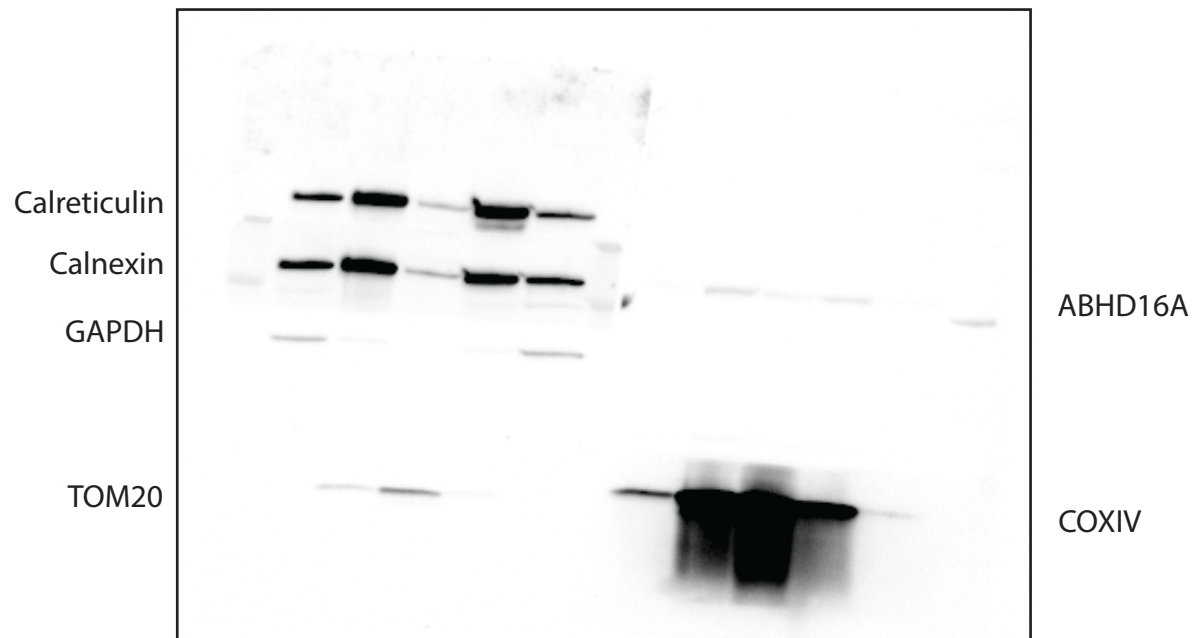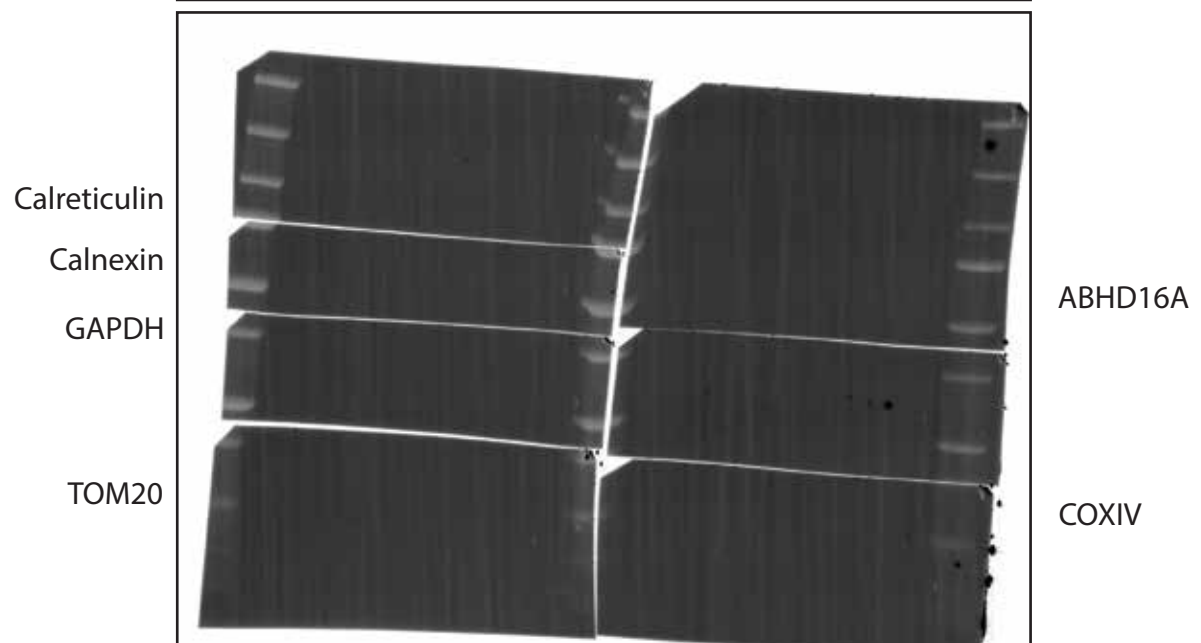

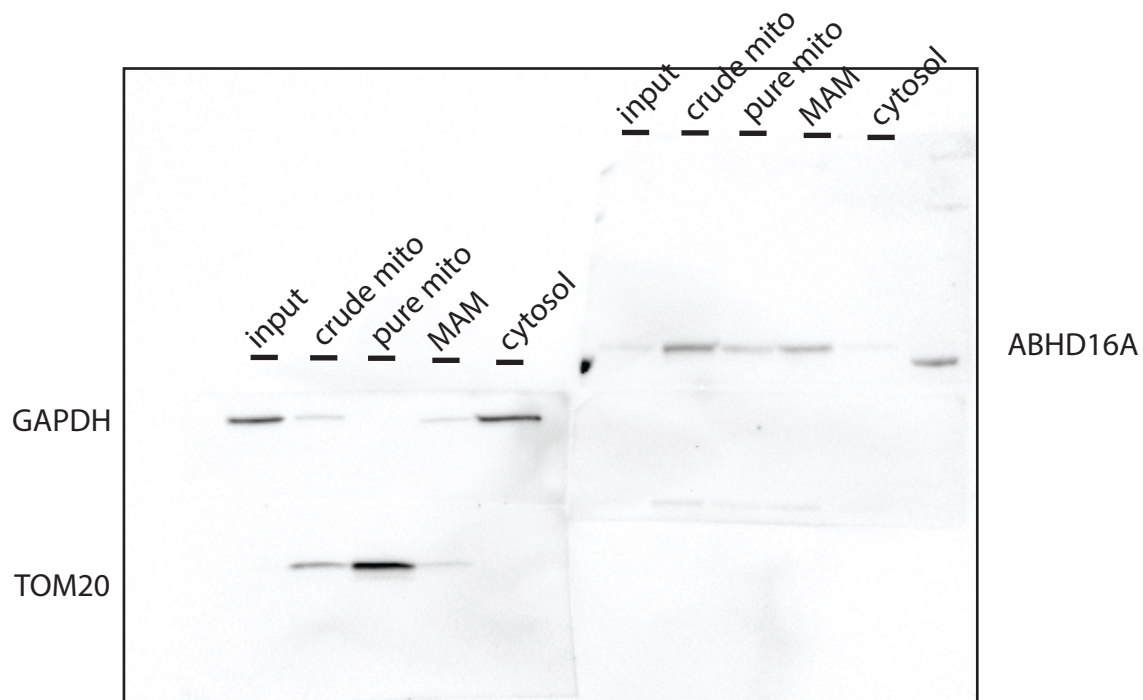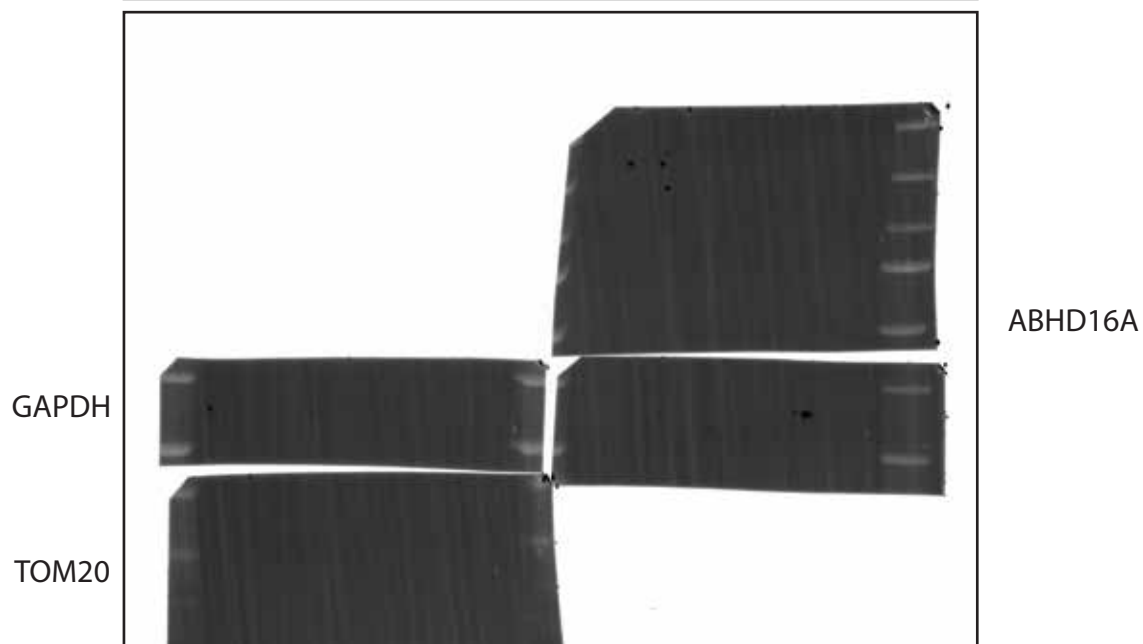

Supplement: Figure 1—figure supplement 1—source data 1. [file elife-84279-fig1-figsupp1-data1.pdf]

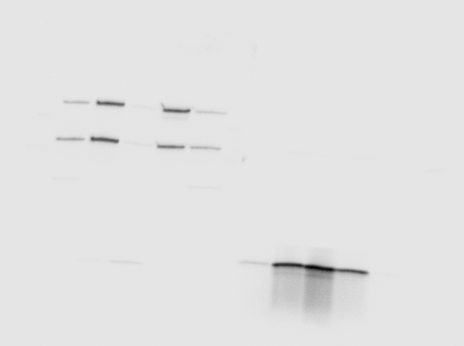

Supplement: Figure 1—figure supplement 1—source data 2. [file elife-84279-fig1-figsupp1-data2.zip › Figure 1-figure supplement 1-source data 2.tif]

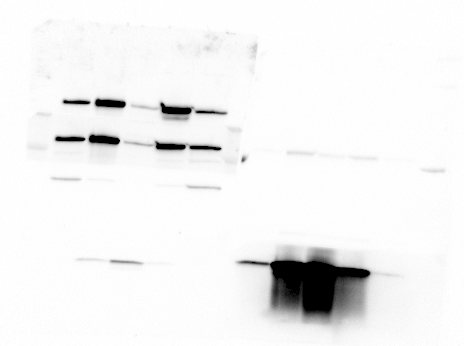

Supplement: Figure 1—figure supplement 1—source data 3. [file elife-84279-fig1-figsupp1-data3.zip › Figure 1-figure supplement 1-source data 3.tif]

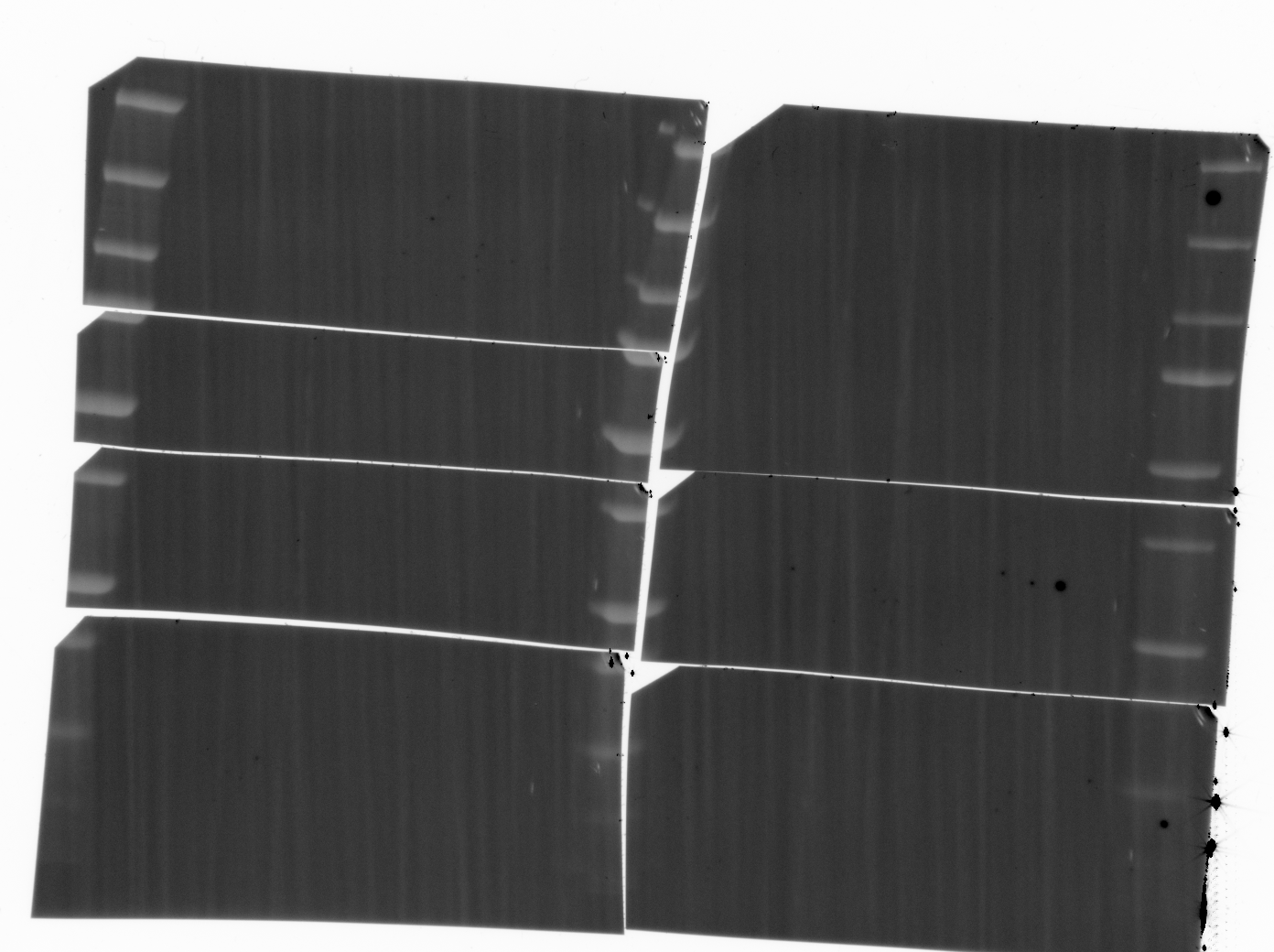

Supplement: Figure 1—figure supplement 1—source data 4. [file elife-84279-fig1-figsupp1-data4.zip › Figure 1-figure supplement 1-source data 4.tif]

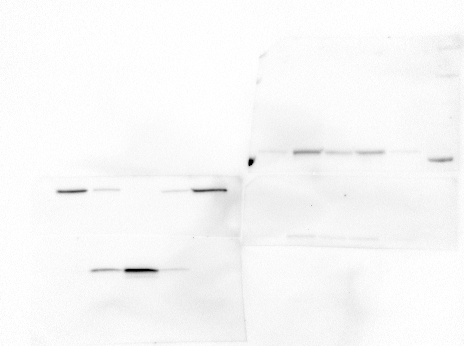

Supplement: Figure 1—figure supplement 1—source data 5. [file elife-84279-fig1-figsupp1-data5.zip › Figure 1-figure supplement 1-source data 5.tif]

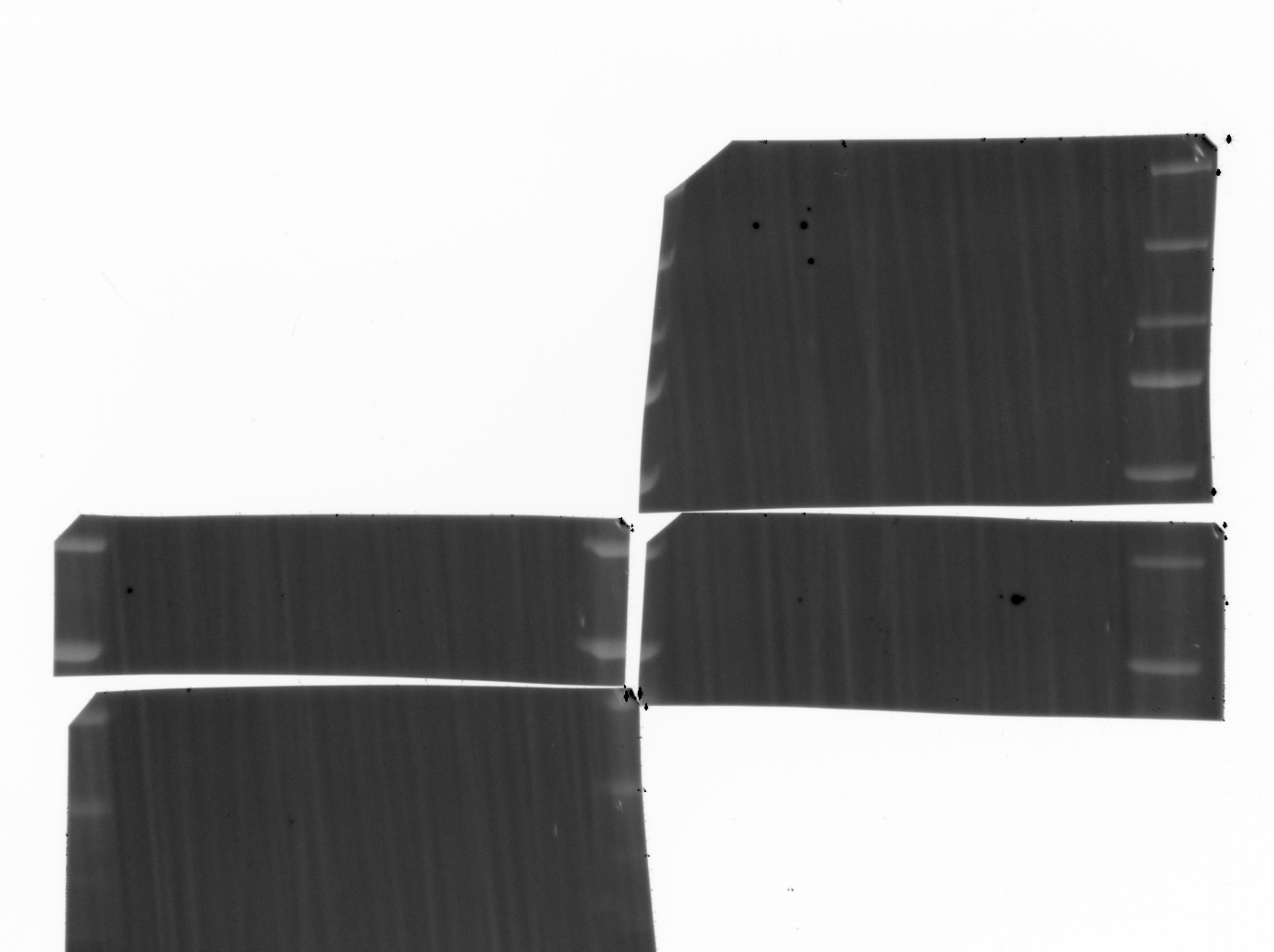

Supplement: Figure 1—figure supplement 1—source data 6. [file elife-84279-fig1-figsupp1-data6.zip › Figure 1-figure supplement 1-source data 6.tif]

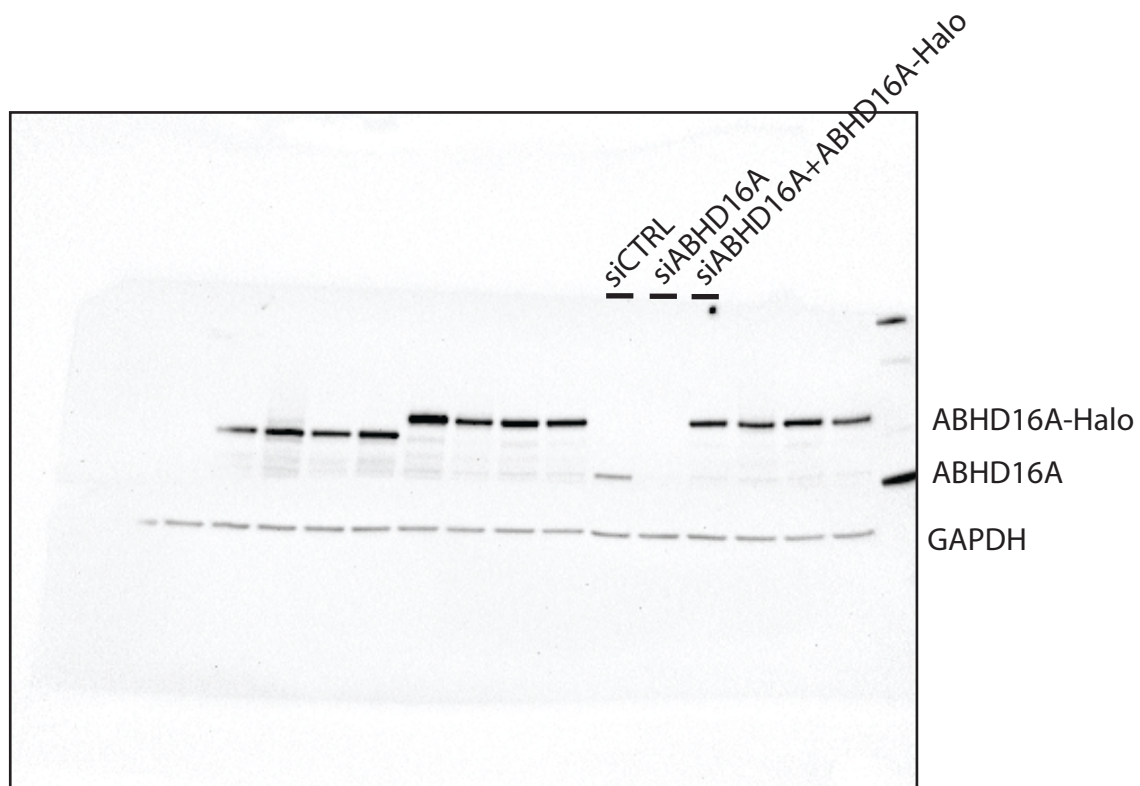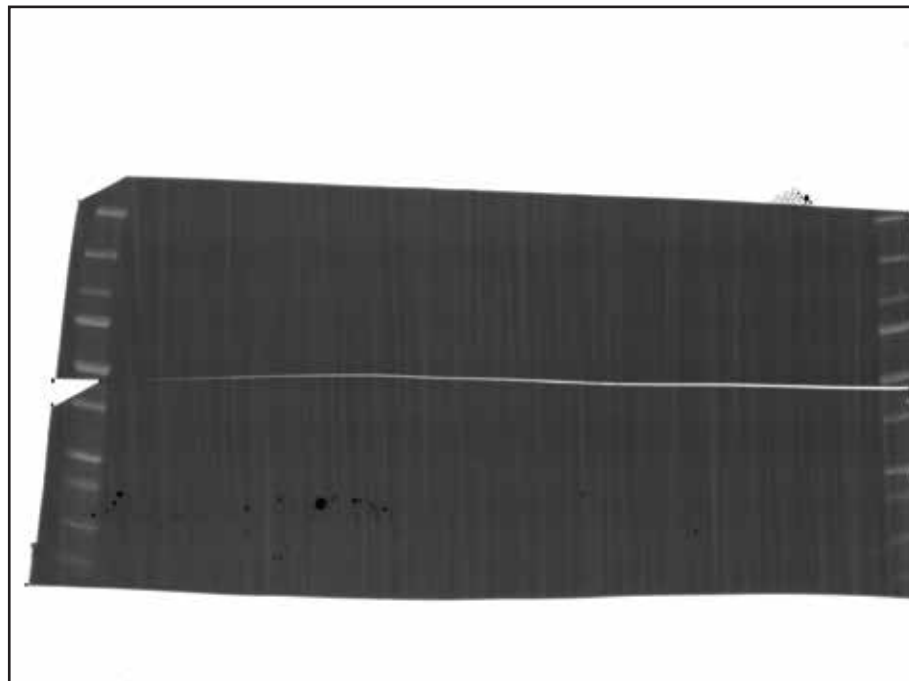

Supplement: Figure 2—figure supplement 1—source data 1. [file elife-84279-fig2-figsupp1-data1.pdf]

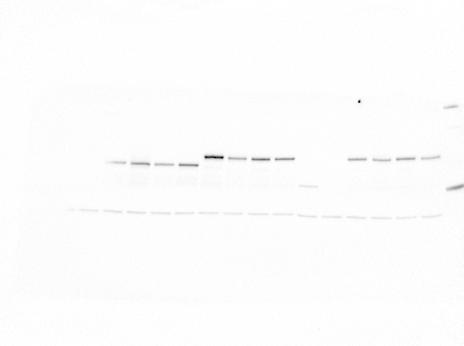

Supplement: Figure 2—figure supplement 1—source data 2. [file elife-84279-fig2-figsupp1-data2.zip › Figure 2-figure supplement 1-source data 2.tif]

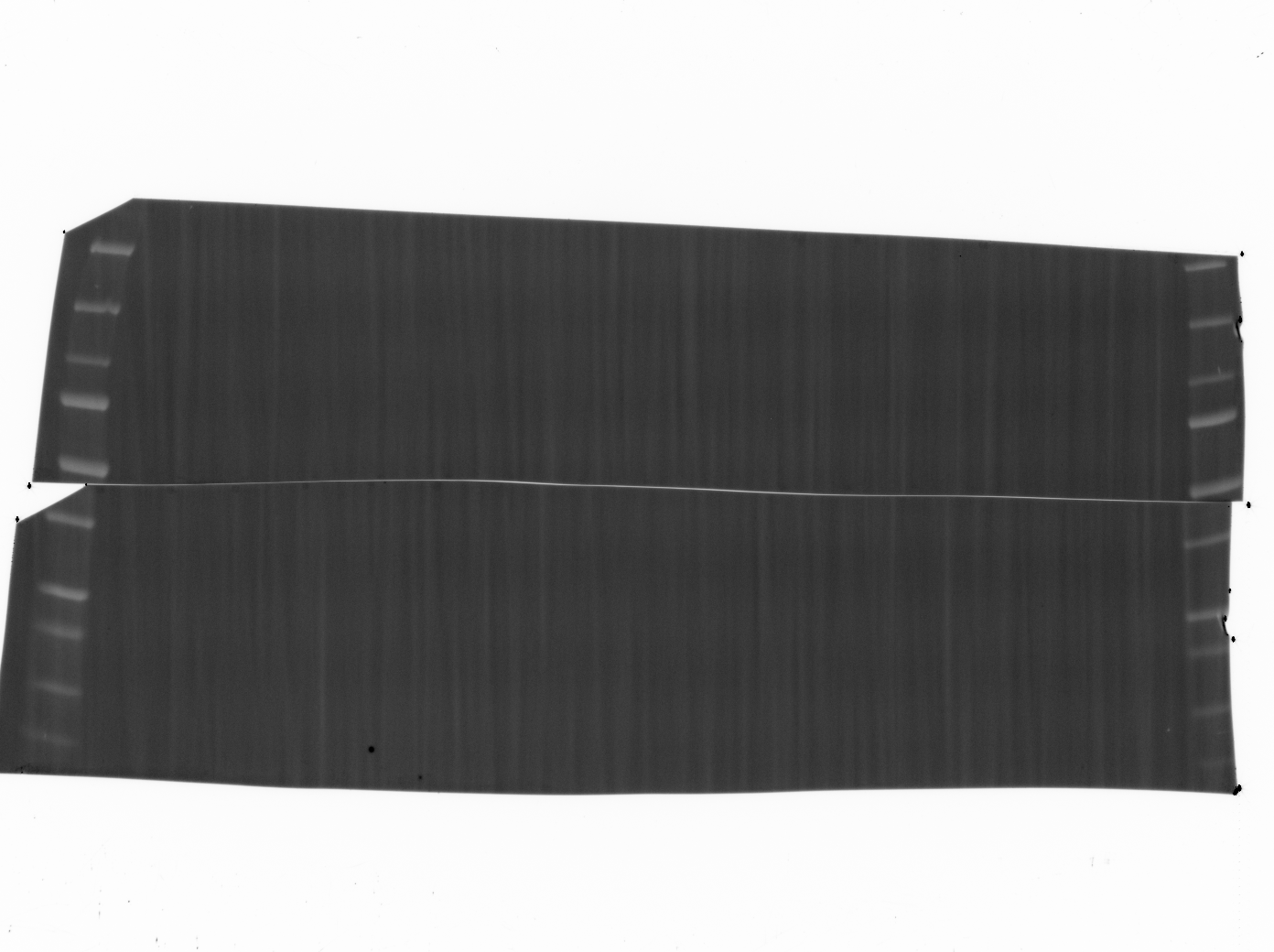

Supplement: Figure 2—figure supplement 1—source data 3. [file elife-84279-fig2-figsupp1-data3.zip › Figure 2-figure supplement 1-source data 3.tif]

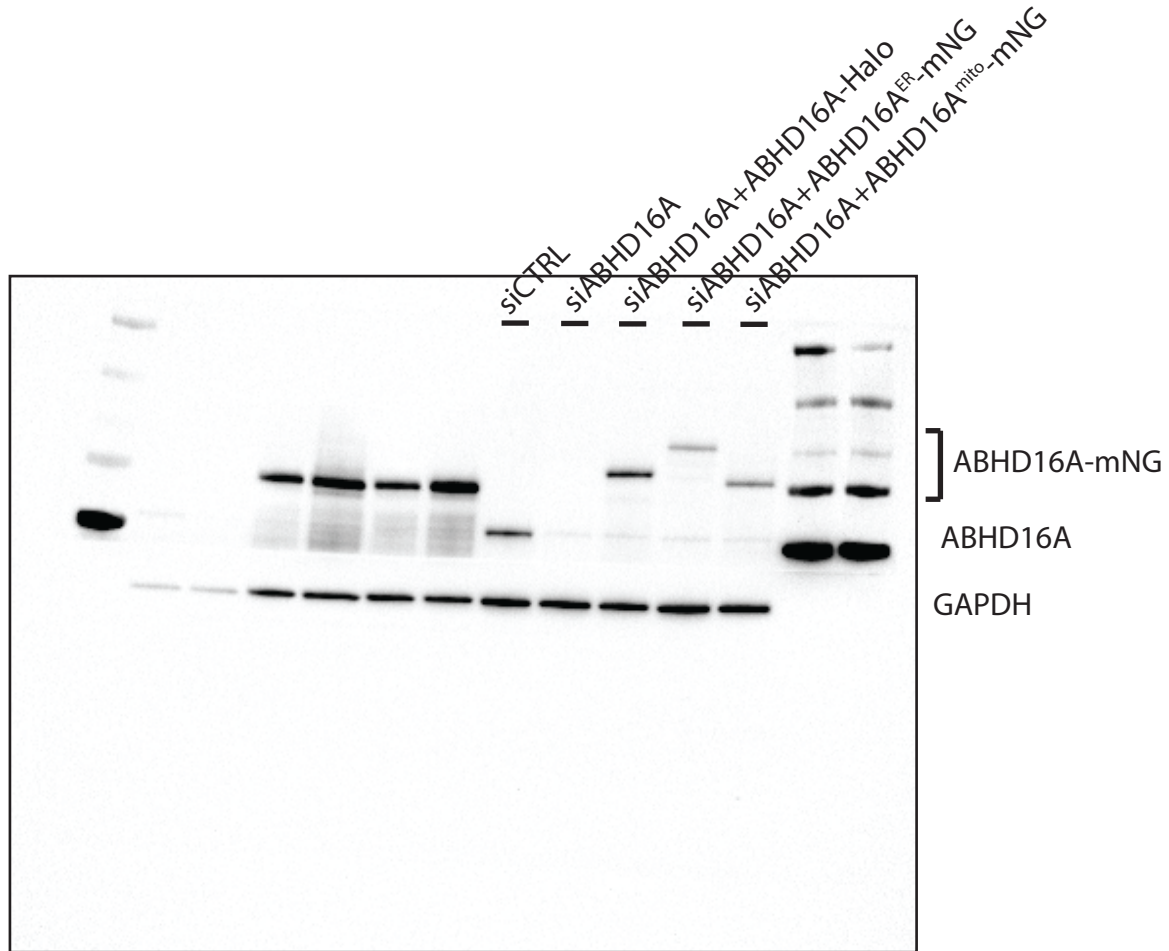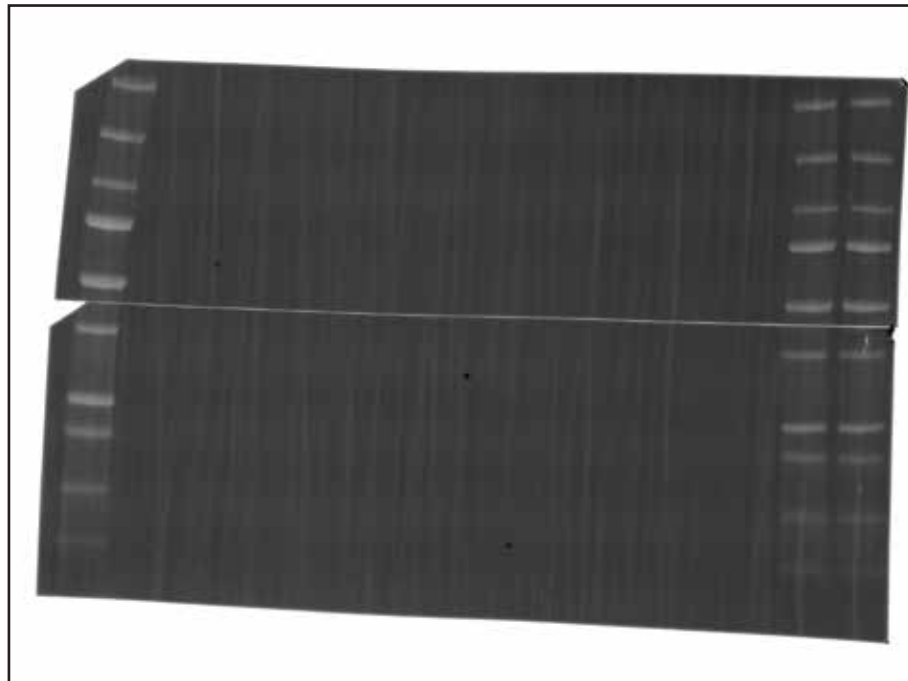

Supplement: Figure 4—source data 2. [file elife-84279-fig4-data2.pdf]

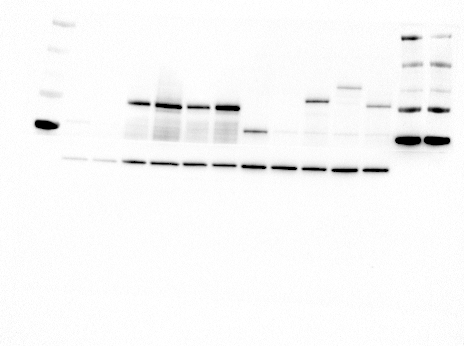

Supplement: Figure 4—source data 3. [file elife-84279-fig4-data3.zip › Figure 4-source data 3.tif]

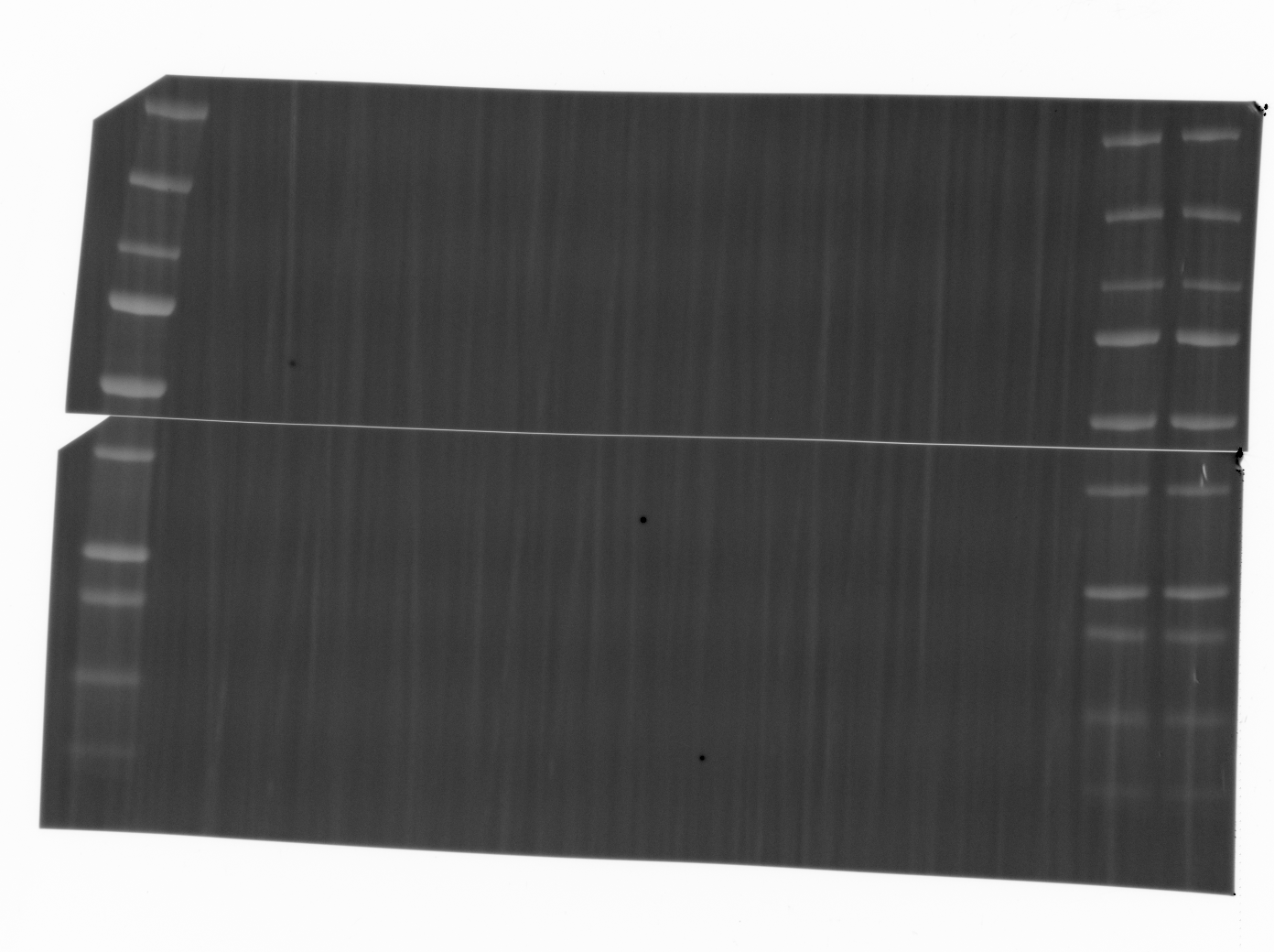

Supplement: Figure 4—source data 4. [file elife-84279-fig4-data4.zip › Figure 4-source data 4.tif]

siCTRL  
siABHD16A  
siABHD16A+ABHD16A-mCh

ABHD16A-mCh

ABHD16A

GAPDH

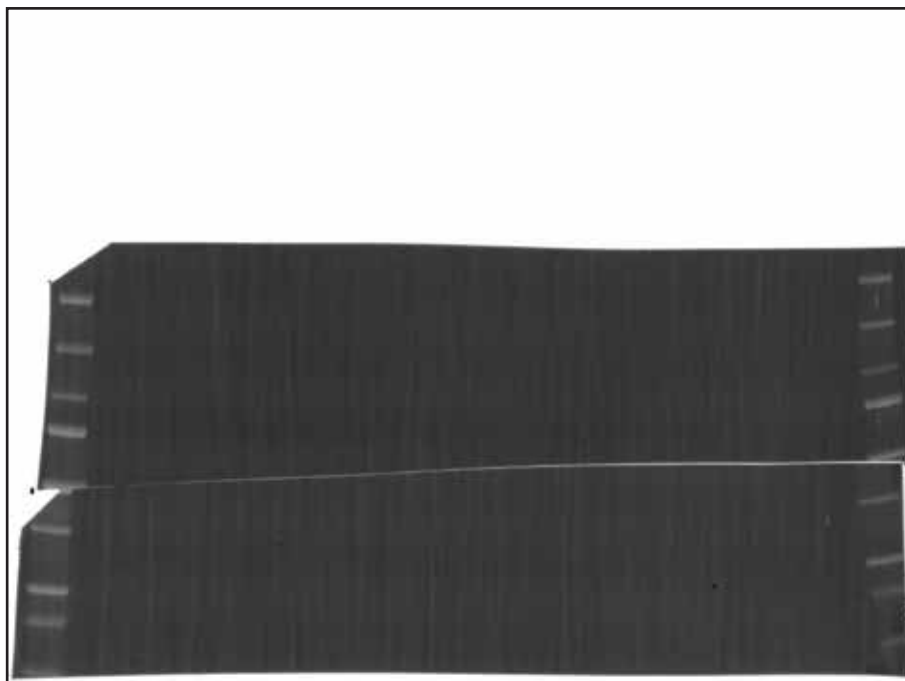

Supplement: Figure 4—figure supplement 1—source data 1. [file elife-84279-fig4-figsupp1-data1.pdf]

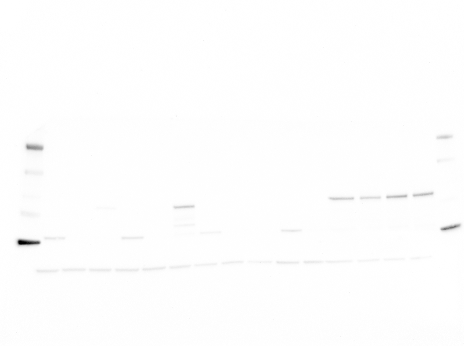

Supplement: Figure 4—figure supplement 1—source data 3. [file elife-84279-fig4-figsupp1-data3.zip › Figure 4-figure supplement 1-source data 3.tif]

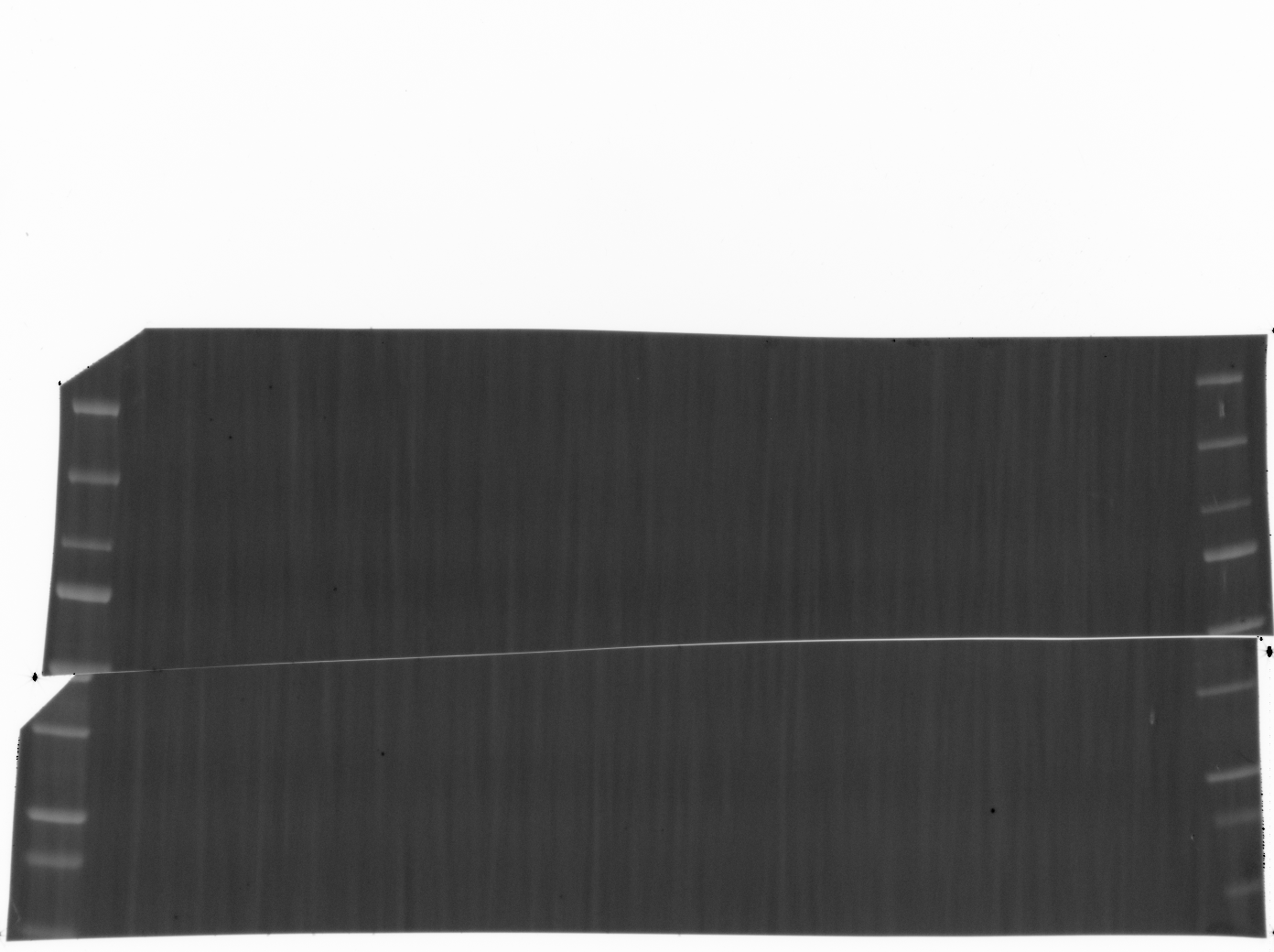

Supplement: Figure 4—figure supplement 1—source data 4. [file elife-84279-fig4-figsupp1-data4.zip › Figure 4-figure supplement 1-source data 4.tif]

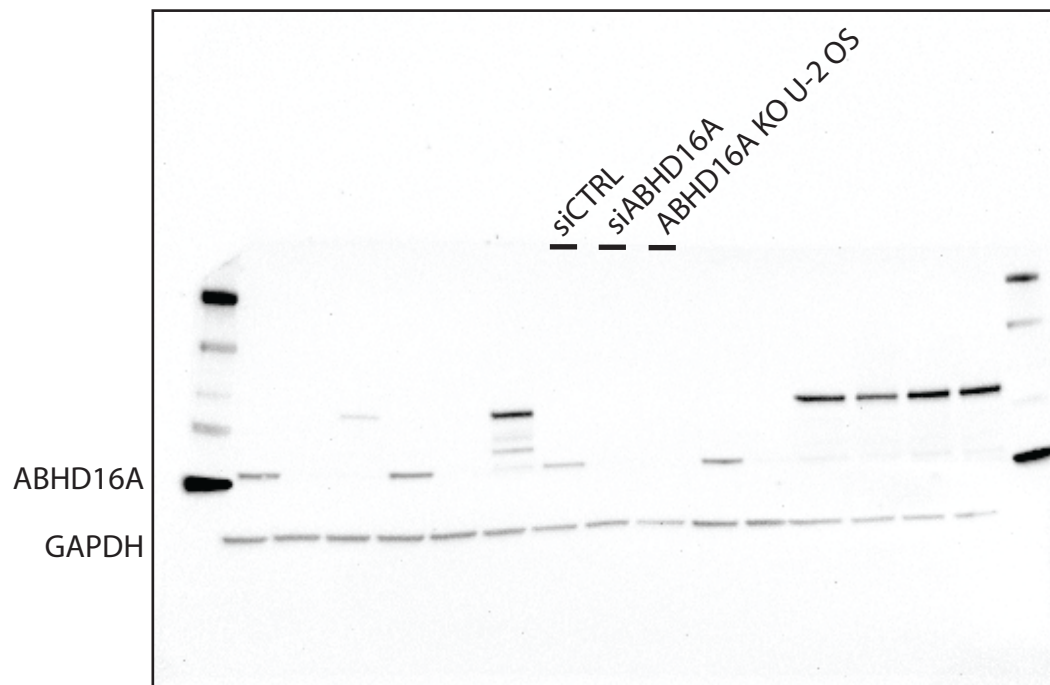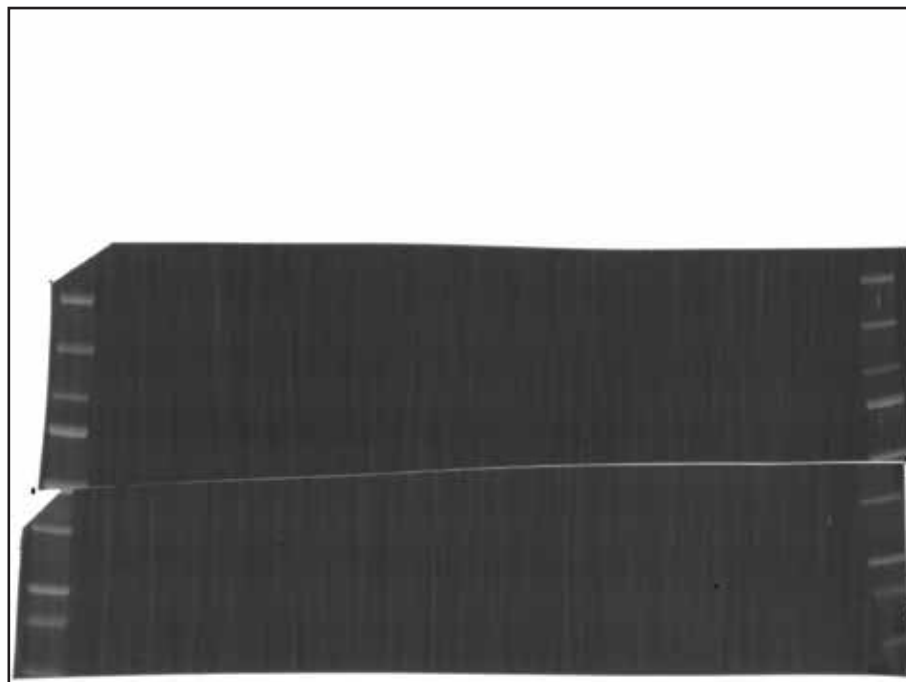

Supplement: Figure 4—figure supplement 1—source data 5. [file elife-84279-fig4-figsupp1-data5.pdf]

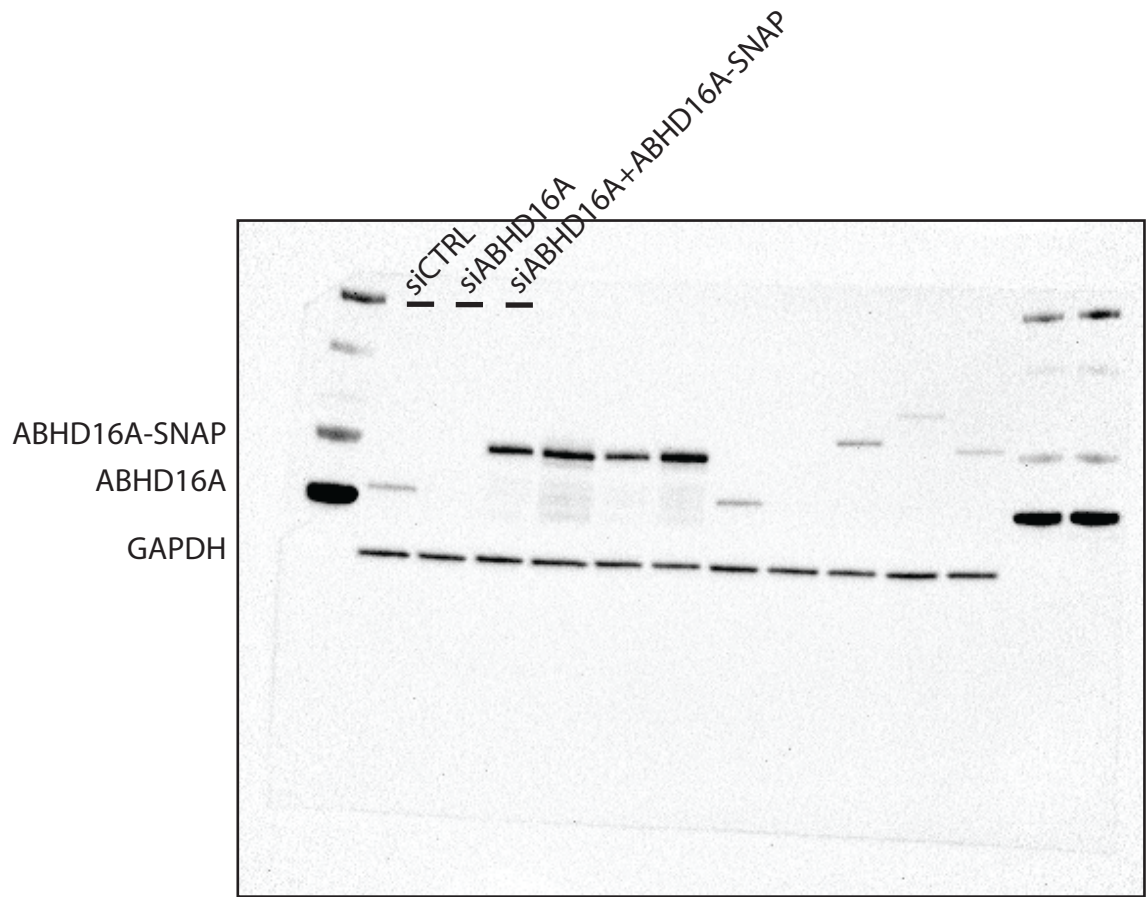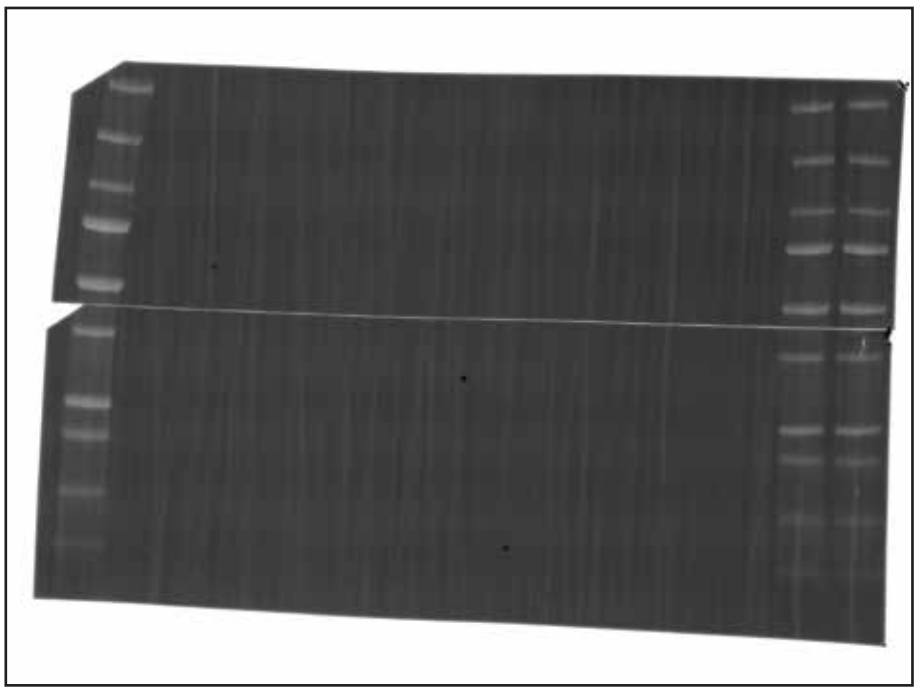

Supplement: Figure 5—figure supplement 1—source data 1. [file elife-84279-fig5-figsupp1-data1.pdf]

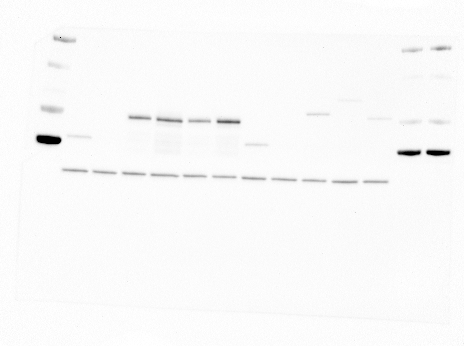

Supplement: Figure 5—figure supplement 1—source data 2. [file elife-84279-fig5-figsupp1-data2.zip › Figure 5-figure supplement 1-source data 2.tif]

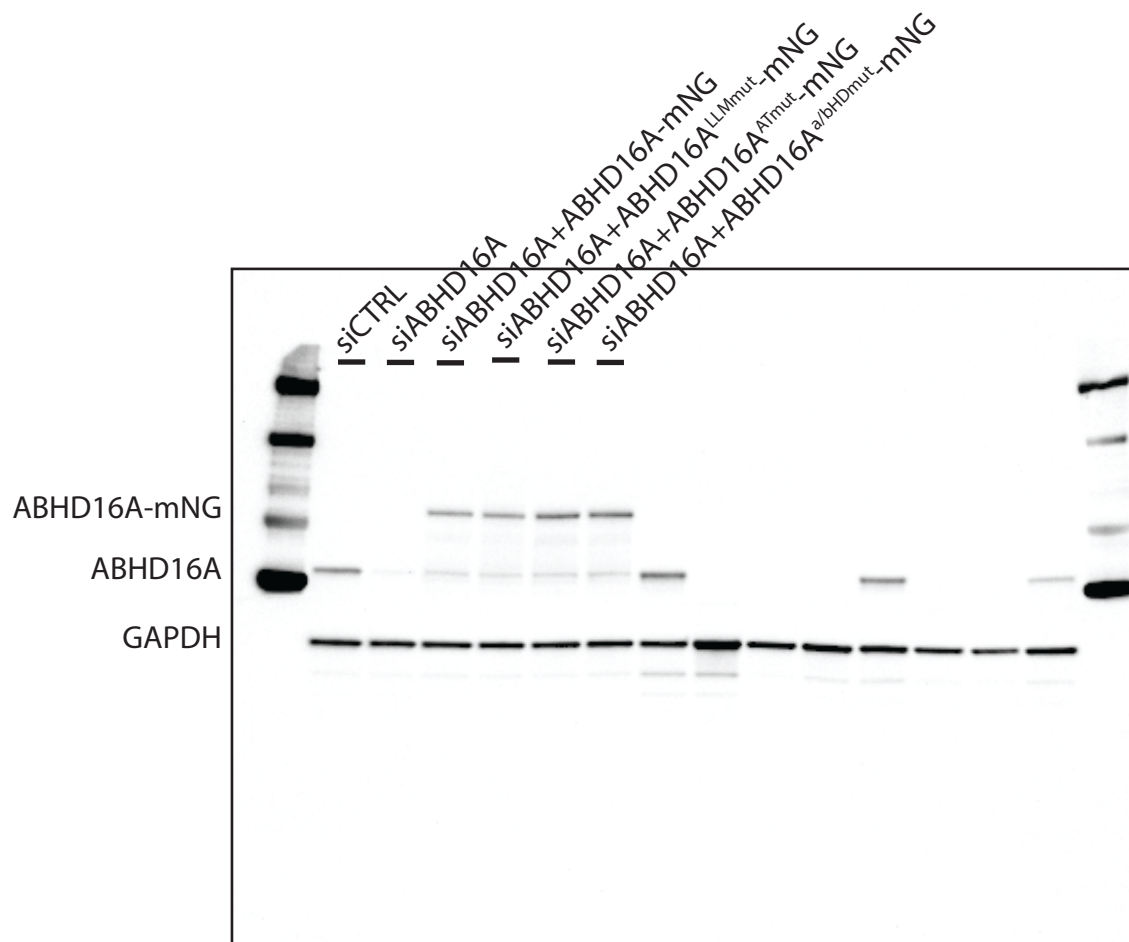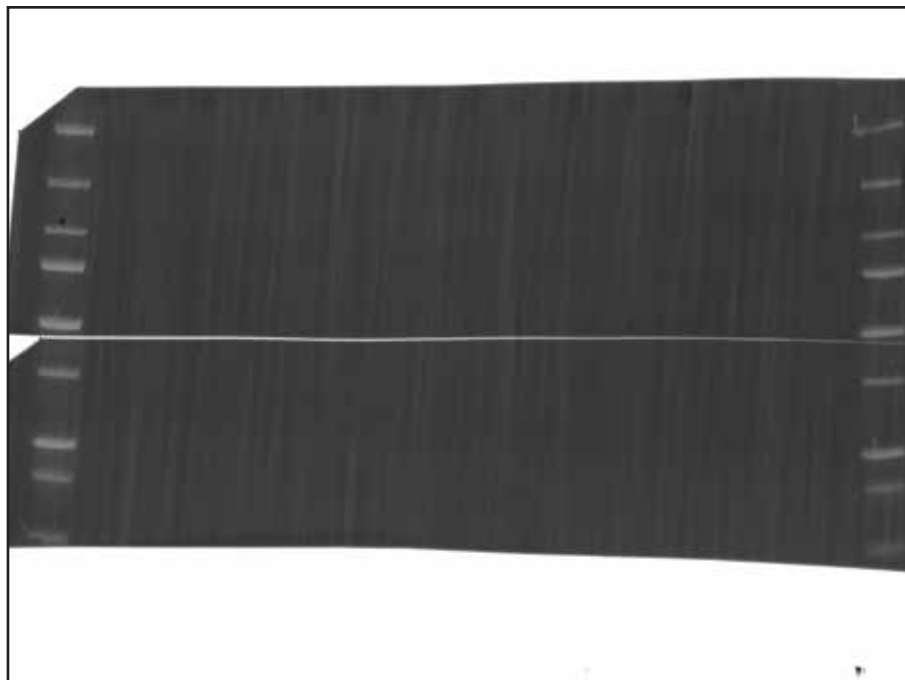

Supplement: Figure 6—figure supplement 1—source data 1. [file elife-84279-fig6-figsupp1-data1.pdf]

ABHD16A-Halo  
ABHD16A  
GAPDH

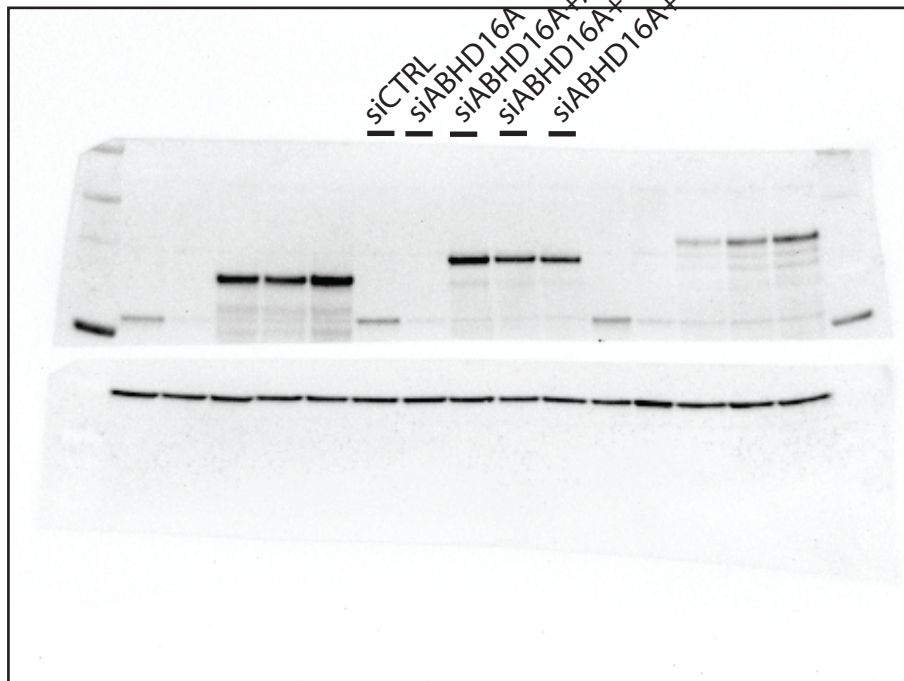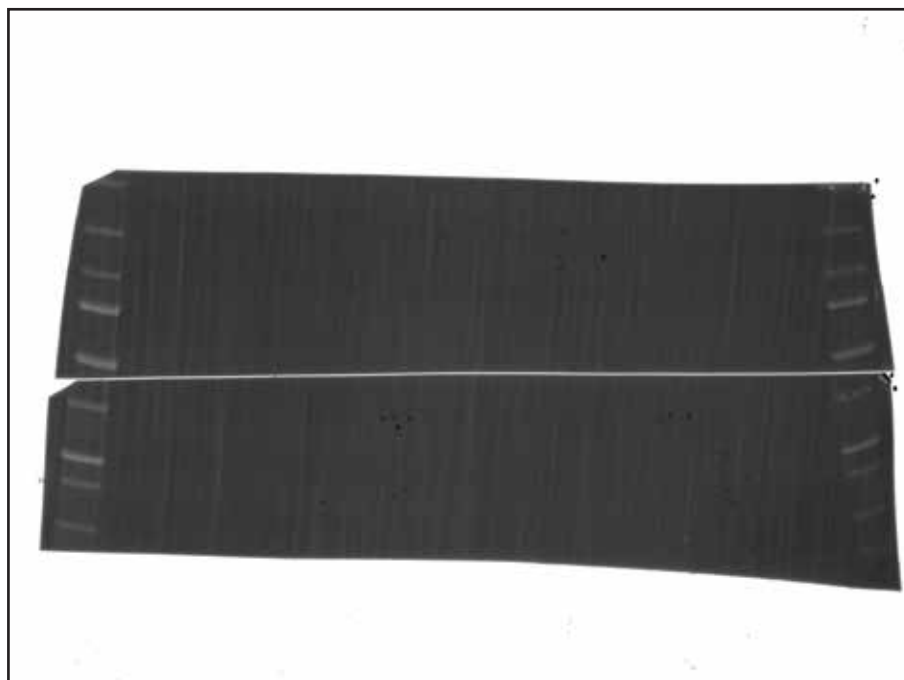

Supplement: Figure 6—figure supplement 1—source data 3. [file elife-84279-fig6-figsupp1-data3.pdf]

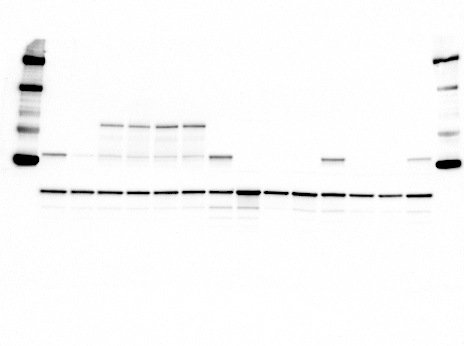

Supplement: Figure 6—figure supplement 1—source data 4. [file elife-84279-fig6-figsupp1-data4.zip › Figure 6-figure supplement 1-source data 4.tif]

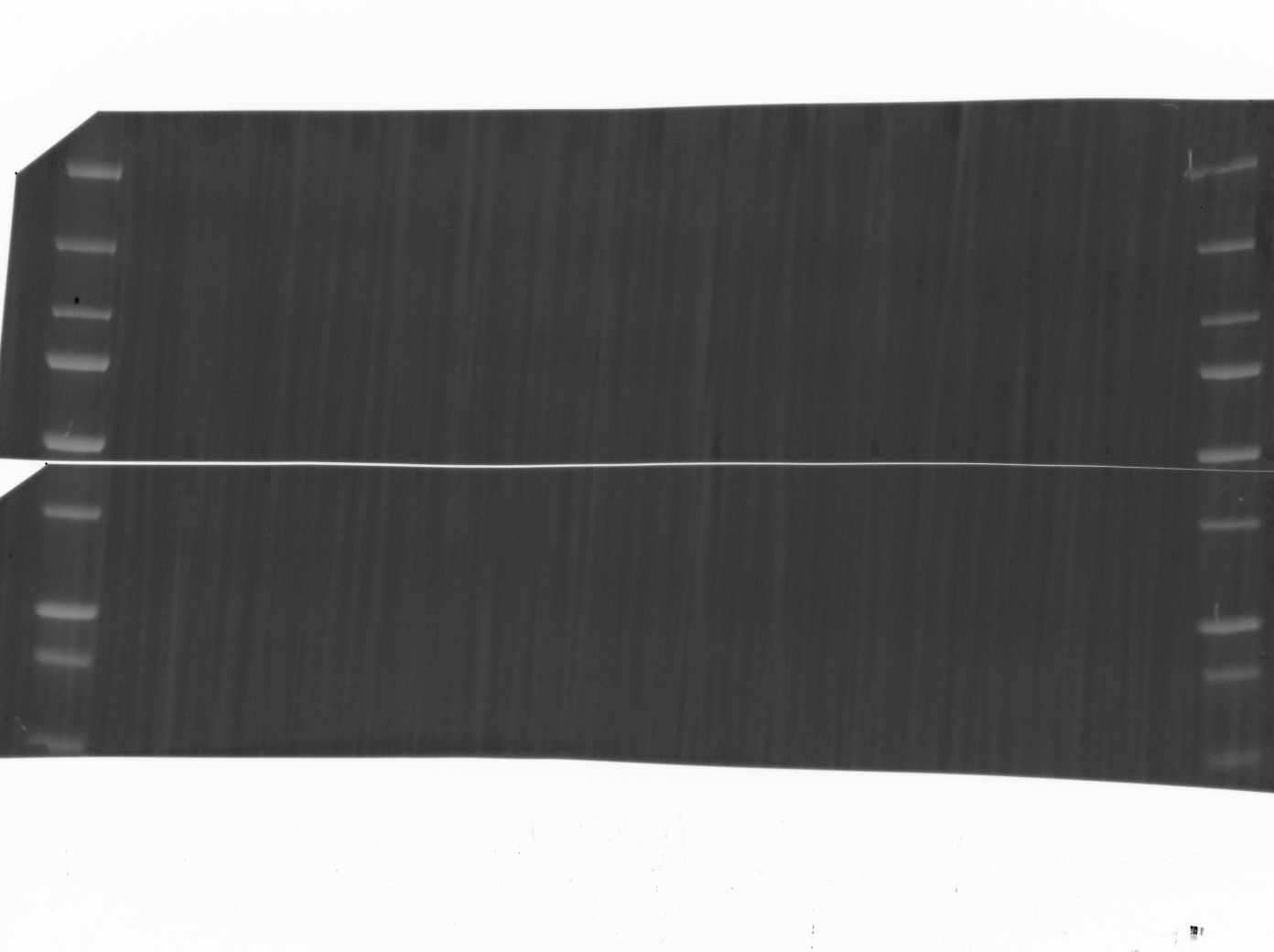

Supplement: Figure 6—figure supplement 1—source data 5. [file elife-84279-fig6-figsupp1-data5.zip › Figure 6-figure supplement 1-source data 5.tif]

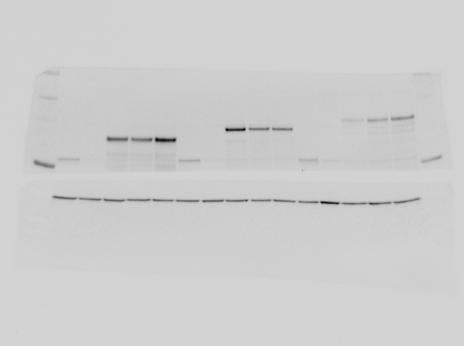

Supplement: Figure 6—figure supplement 1—source data 6. [file elife-84279-fig6-figsupp1-data6.zip › Figure 6-figure supplement 1-source data 6.tif]

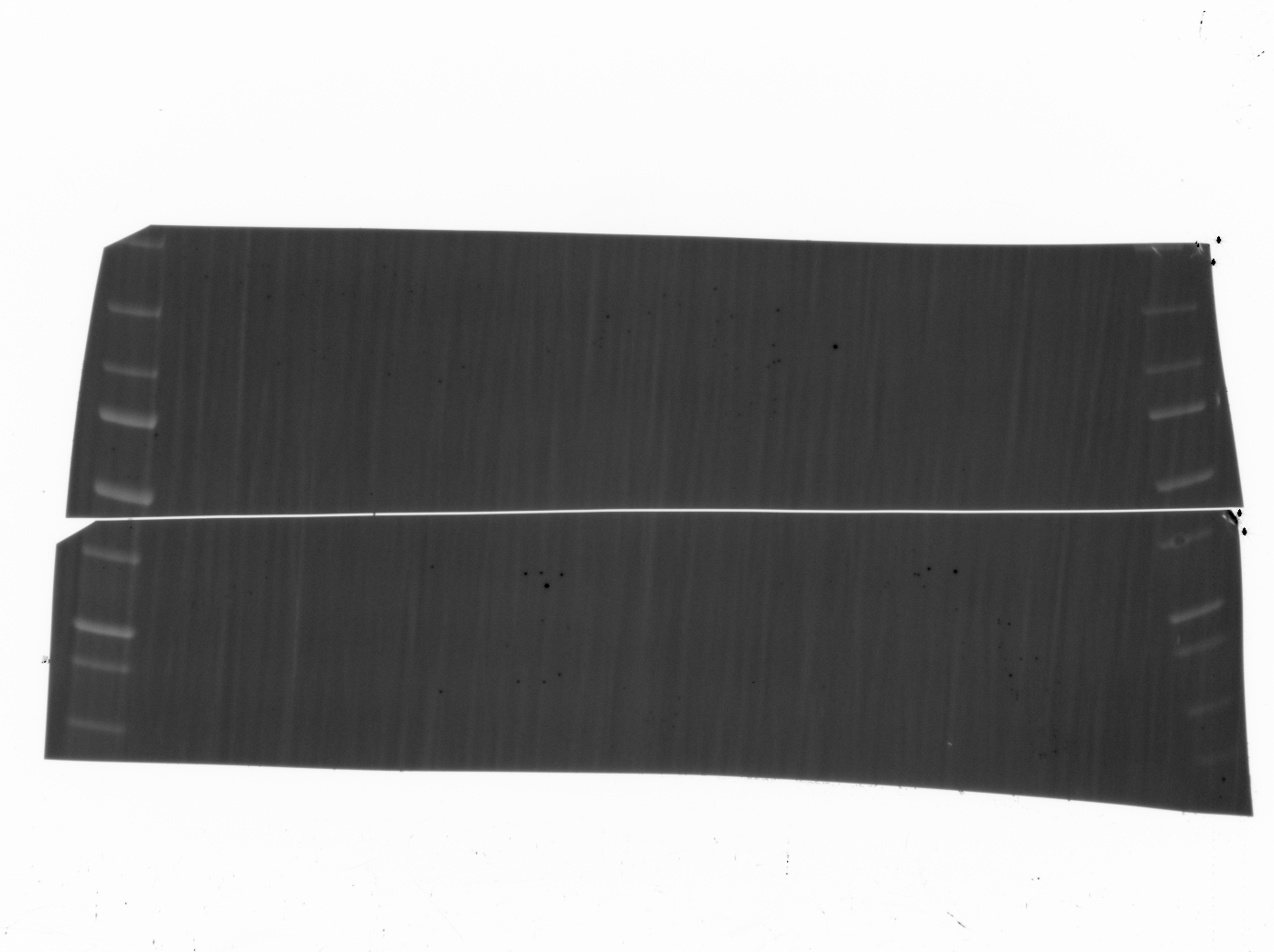

Supplement: Figure 6—figure supplement 1—source data 7. [file elife-84279-fig6-figsupp1-data7.zip › Figure 6-figure supplement 1-source data 7.tif]

GAPDH

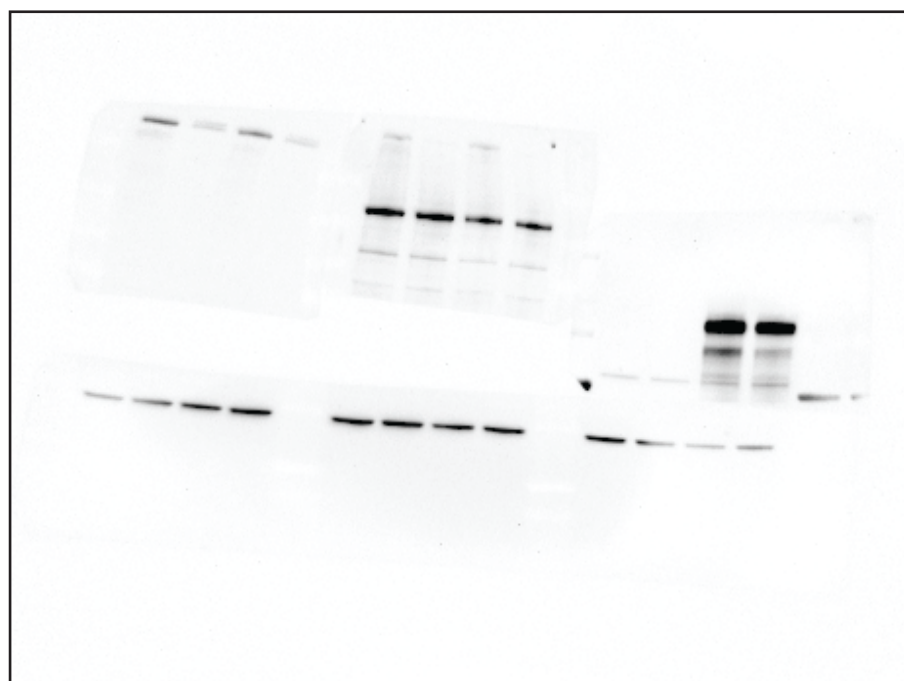

ABHD16A-mCh  
ABHD16A  
GAPDH

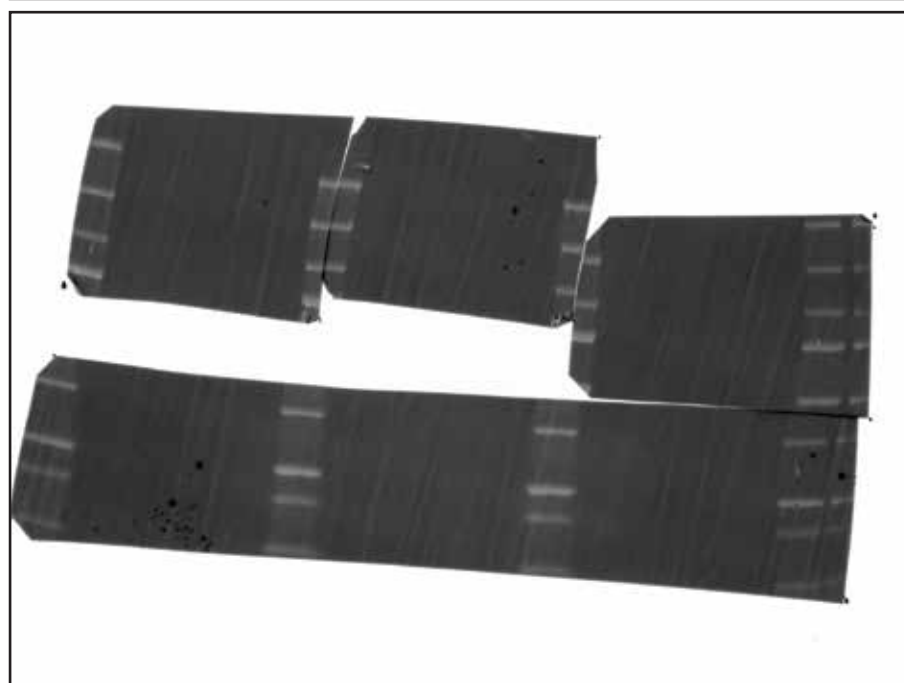

Supplement: Figure 7—source data 3. [file elife-84279-fig7-data3.pdf]

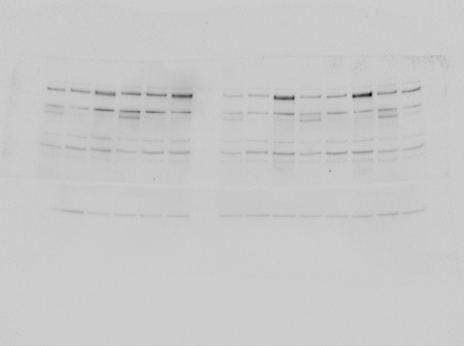

Supplement: Figure 7—source data 4. [file elife-84279-fig7-data4.zip › Figure 7 source data 4.tif]

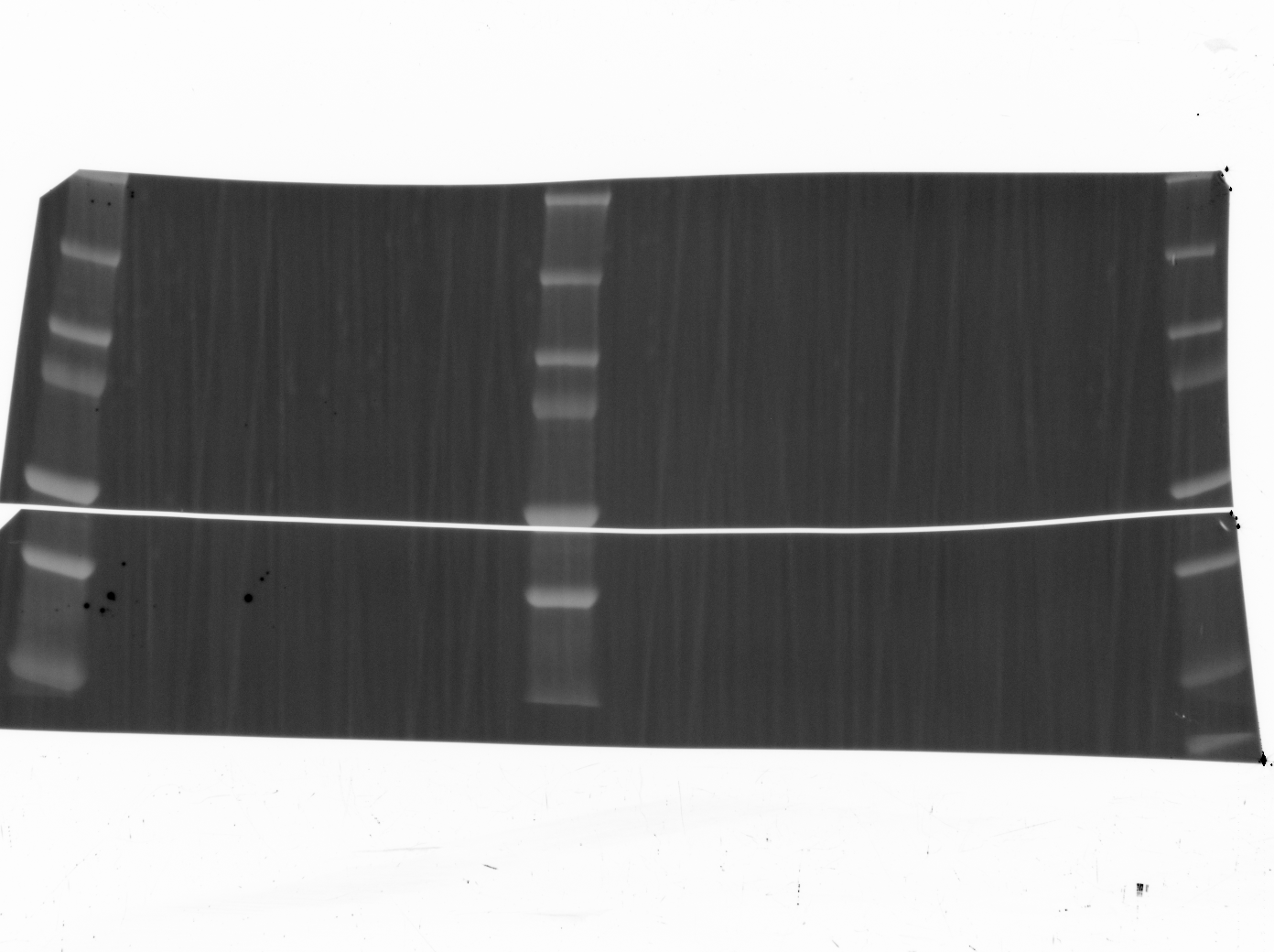

Supplement: Figure 7—source data 5. [file elife-84279-fig7-data5.zip › Figure 7 source data 5.tif]

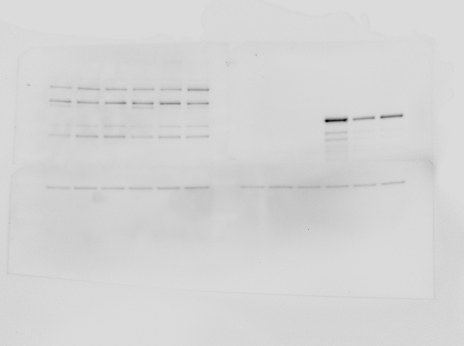

Supplement: Figure 7—source data 6. [file elife-84279-fig7-data6.zip › Figure 7 source data 6.tif]

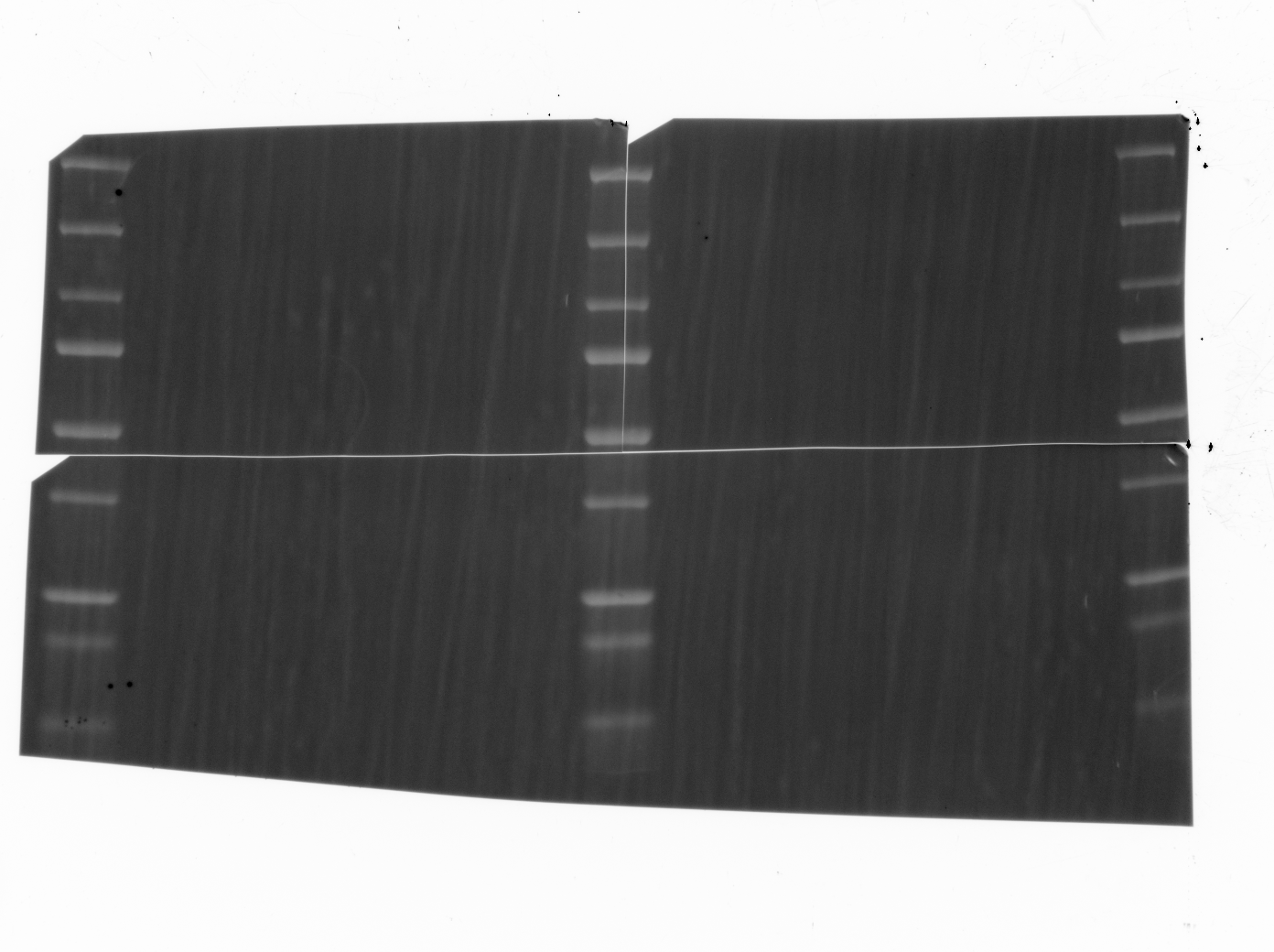

Supplement: Figure 7—source data 7. [file elife-84279-fig7-data7.zip › Figure 7 source data 7.tif]

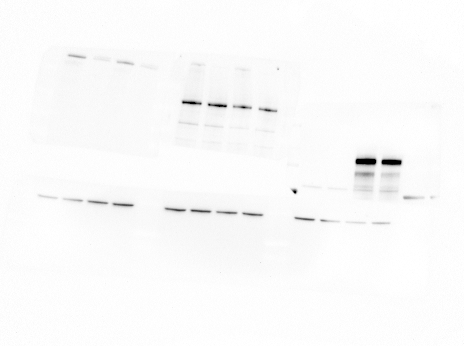

Supplement: Figure 7—source data 8. [file elife-84279-fig7-data8.zip › Figure 7 source data 8.tif]

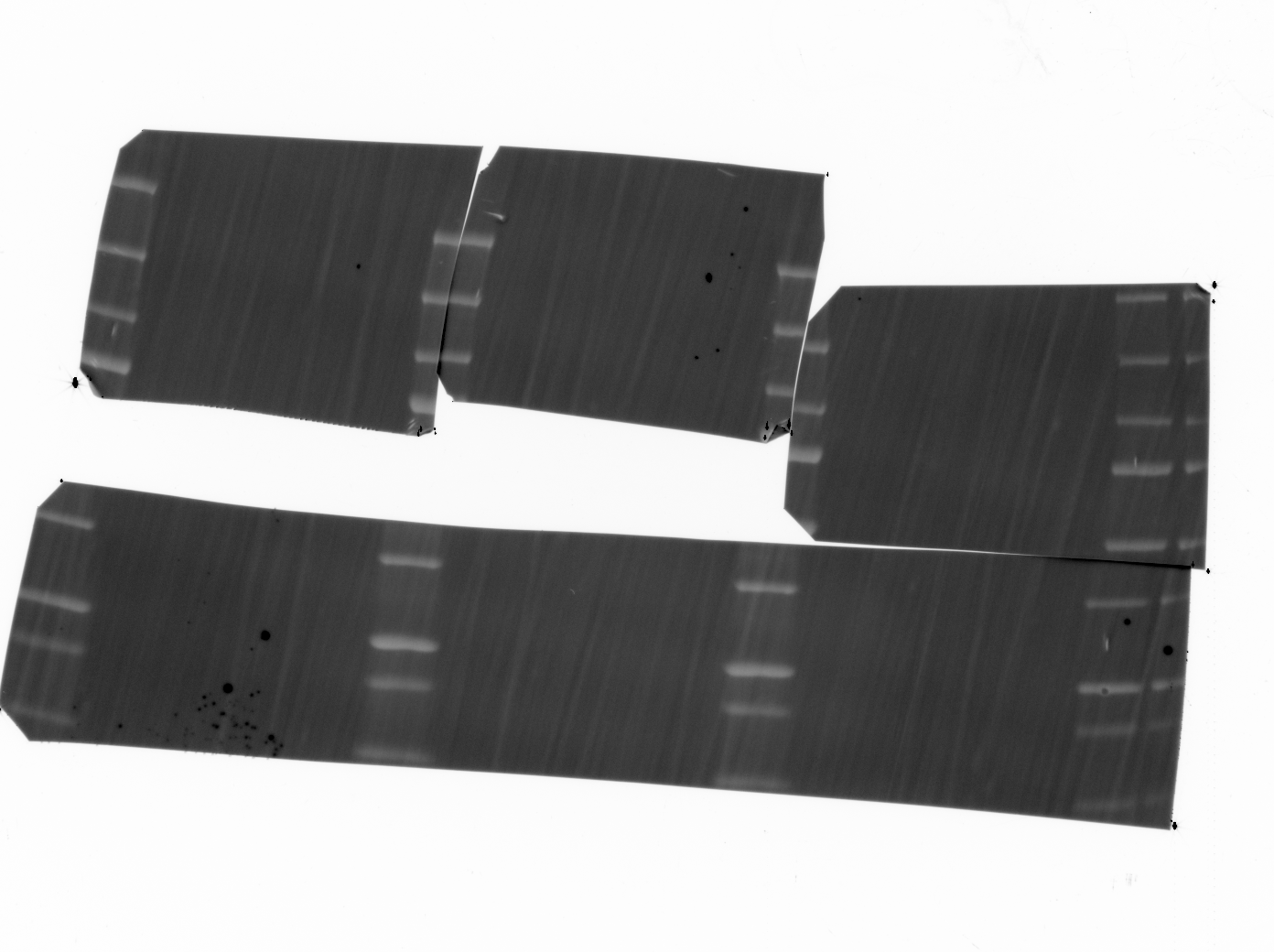

Supplement: Figure 7—source data 9. [file elife-84279-fig7-data9.zip › Figure 7 source data 9.tif]
