# Supplementary material for: An ER phospholipid hydrolase drives ER-associated mitochondrial constriction for fission and fusion
Source: eLife. 2022 Nov 30;11:e84279. doi: 10.7554/eLife.84279 (PMC9725753; doi:10.7554/eLife.84279)

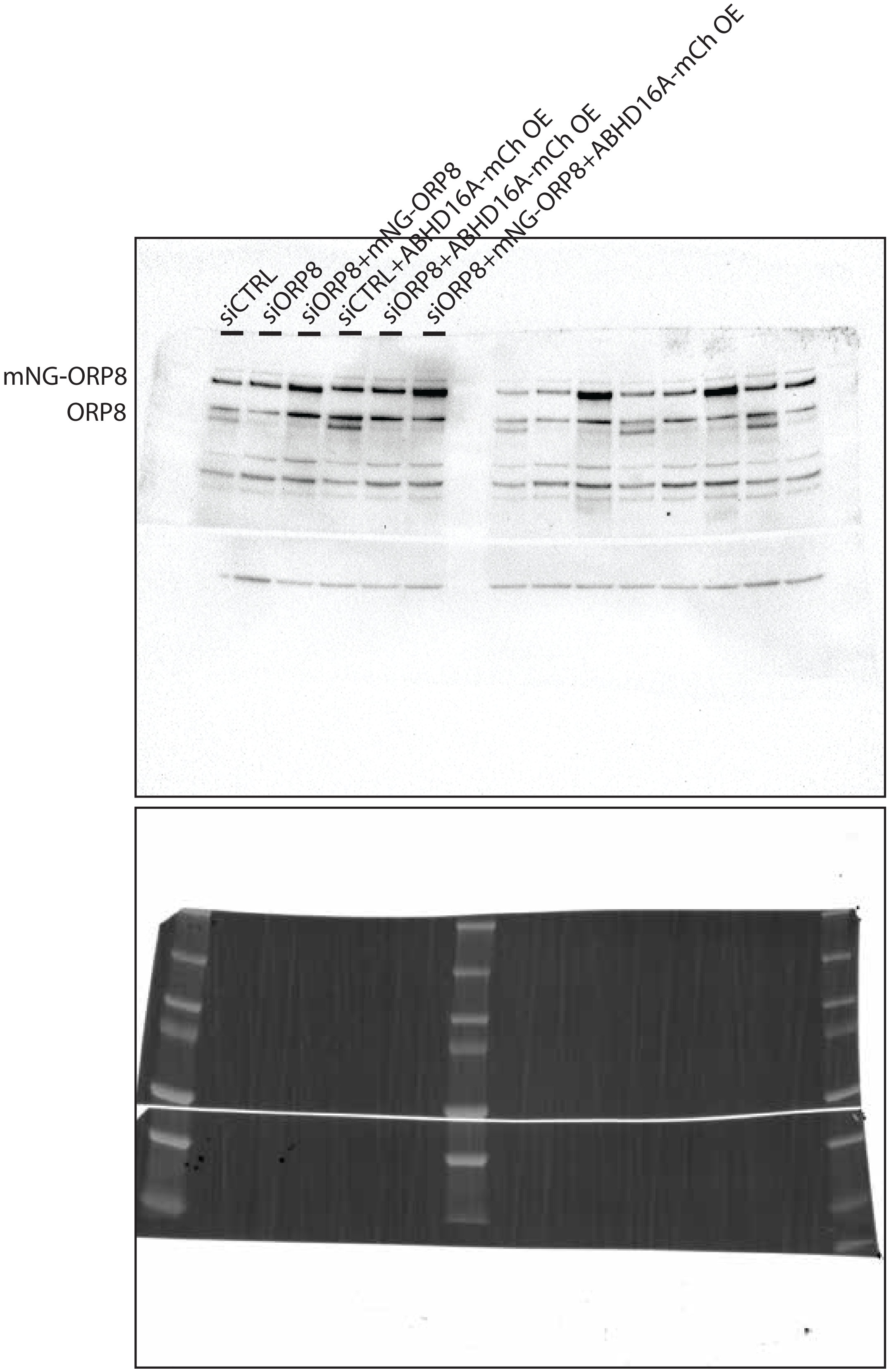
siCTRL
siORP8
siORP8+mNG-ORP8
siCTRL+ABHD16A-mCh OE
siORP8+ABHD16A-mCh OE
siORP8+mNG-ORP8+ABHD16A-mCh OE
mNG-ORP8
ORP8

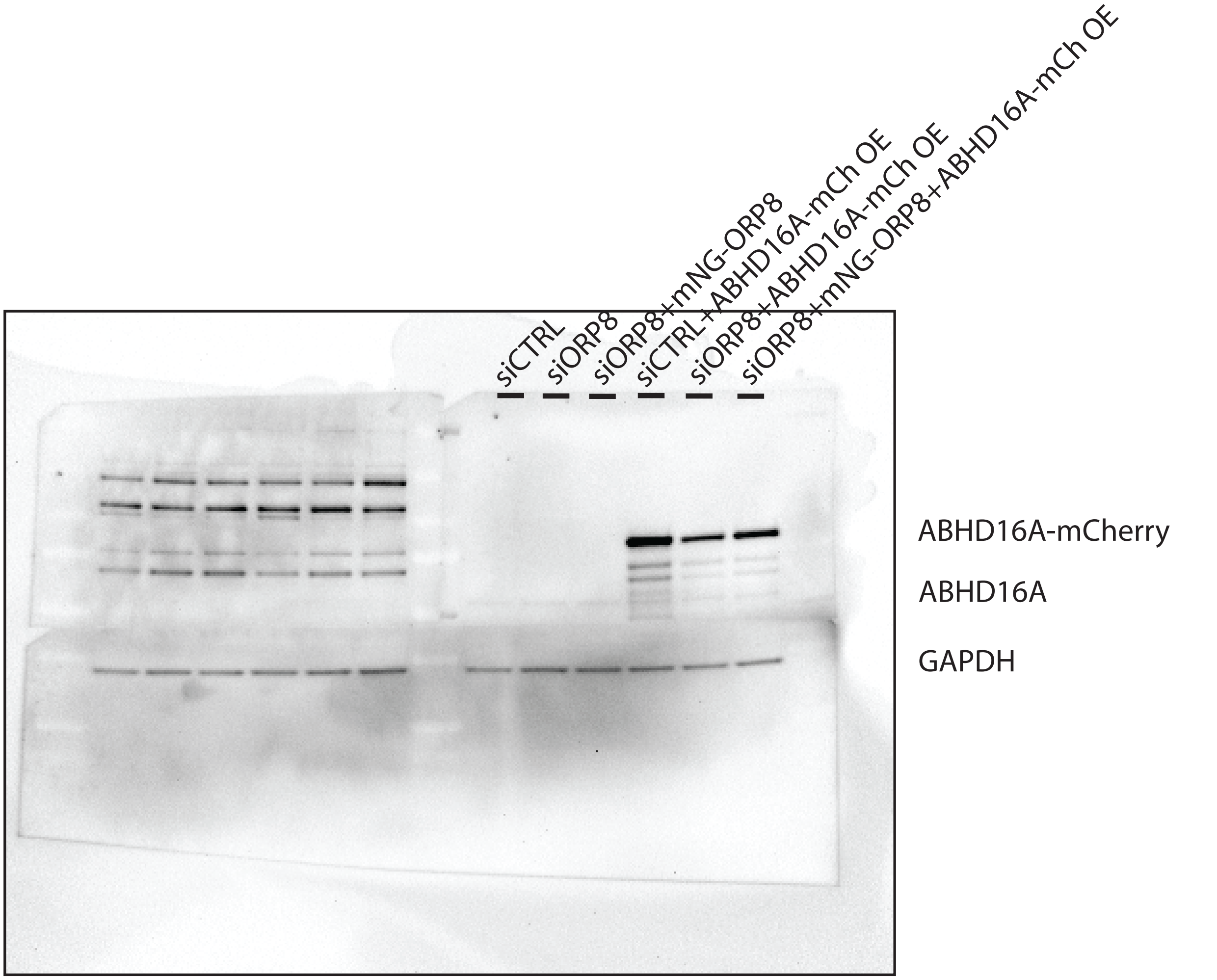

siCTRL
siORP8
siORP8+mNG-ORP8
siCTRL+ABHD16A-mCh OE
siORP8+ABHD16A-mCh OE
siORP8+mNG-ORP8+ABHD16A-mCh OE
ABHD16A-mCherry
ABHD16A
GAPDH

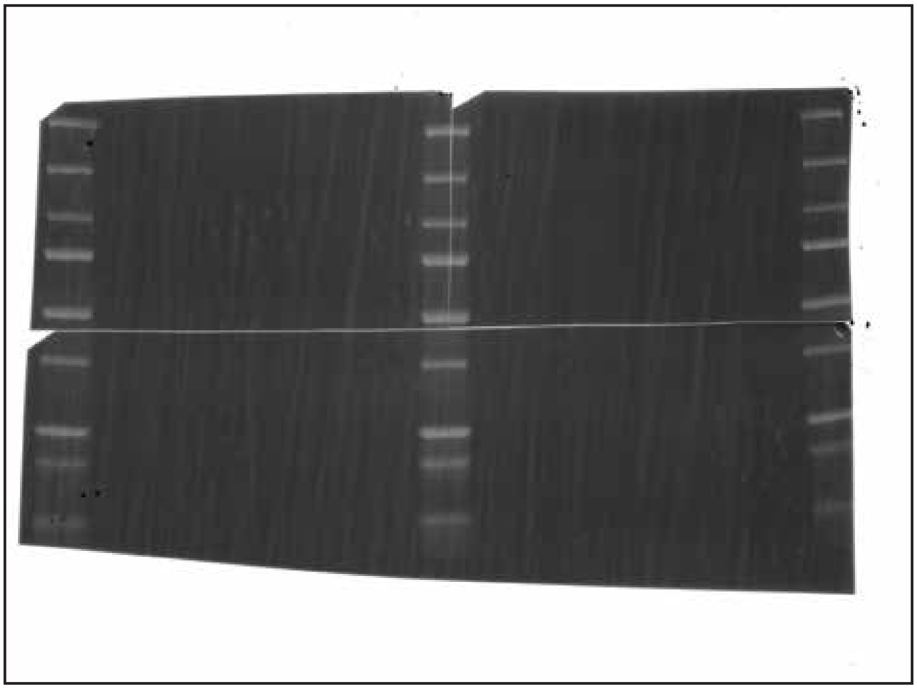

Supplement: Figure 7—source data 2. [file elife-84279-fig7-data2.pdf]
